# FAM117B promotes gastric cancer growth and drug resistance by targeting the KEAP1/NRF2 signaling pathway

**DOI:** 10.1172/JCI158705

**Published:** 2023-02-01

**Authors:** Yunjiang Zhou, Yaxin Chen, Yongwei Shi, Leyin Wu, Yingying Tan, Tao Li, Yigang Chen, Jiazeng Xia, Rong Hu

**Affiliations:** 1 State Key Laboratory of Natural Medicines, School of Basic Medicine and Clinical Pharmacy, China Pharmaceutical University, Nanjing, China.; 2 Department of General Surgery, The Affiliated Wuxi No. 2 People’s Hospital of Nanjing Medical University, Wuxi, China.

**Keywords:** Gastroenterology, Oncology, Cancer, Gastric cancer

## Abstract

Gastric cancer often shows malignant growth and insensitivity to chemotherapeutic drugs due to the regulation of complex molecular mechanisms, which results in poor prognosis for patients. However, the relevant molecular mechanisms remain unclear. In this study, we reported that family with sequence similarity 117, member B (FAM117B), promoted the growth of gastric cancer cells and reduced the sensitivity of cells to chemotherapeutic drugs. Mechanistically, FAM117B competed with nuclear factor E2–related factor 2 (NRF2) for Kelch-like ECH-associated protein 1 (KEAP1) binding, reduced the ubiquitination degradation of NRF2, and activated the KEAP1/NRF2 signaling pathway. Moreover, FAM117B-induced growth and chemoresistance of gastric cancer cells were NRF2 dependent. We found that FAM117B and NRF2 protein levels were highly expressed in tumor tissues of patients with gastric cancer and their co-overexpression represented an independent factor for poor prognosis. Collectively, our findings reveal that FAM117B is involved in promoting gastric cancer growth and drug resistance, and it could be exploited as a cancer therapeutic target.

## Graphical abstract

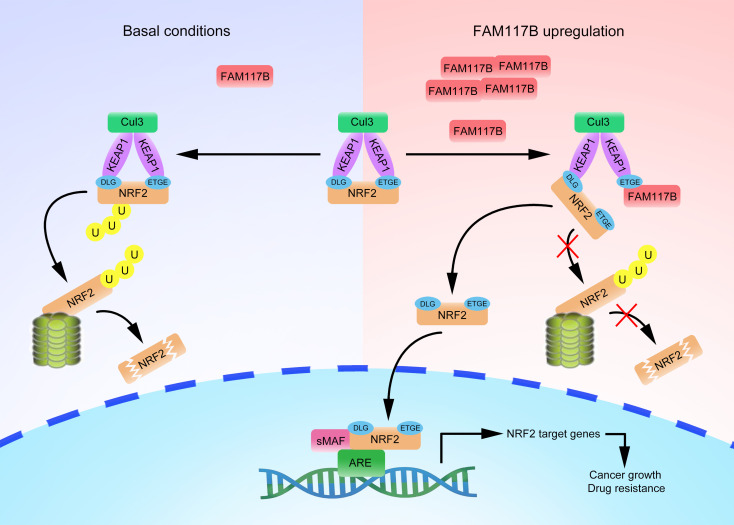

## Introduction

Gastric cancer originates from gastric mucosa epithelial cells and is one of the most common malignant tumors of the digestive system. In 2020, there were about 27,600 new cases of and 11,010 deaths from gastric cancer in the United States (1). Due to the complex molecular mechanisms, gastric cancer often shows malignant growth and resistance to chemotherapeutic agents, which results in poor prognosis for patients. Therefore, it is worthwhile to investigate relevant molecular mechanisms and look for effective therapeutic targets for gastric cancer.

Nuclear factor E2–related factor 2 (NRF2), a transcription factor, participates in regulating cell resistance to oxidative damage. Under normal conditions, NRF2 binds to the DGR domain of Kelch-like ECH-associated protein 1 (KEAP1) through its ETGE and DLG motif in the cytoplasm, which results in its degradation by the proteasome (2–4). Therefore, NRF2 protein is maintained at a low level in the cytoplasm. Under oxidative or electrophilic pressure, covalent modification of certain cysteine residues of KEAP1 destroys the weak binding between KEAP1 and NRF2. Subsequently, KEAP1-mediated NRF2 degradation is blocked and intracellular free NRF2 increases (5). Free NRF2 in the cytoplasm transfers to the nucleus, forms heterodimers with small musculoaponeurotic fibrosarcoma proteins, and then promotes the transcription of NRF2 target genes. Previous studies have shown that overactivation of the KEAP1/NRF2 signaling pathway can promote the growth and chemoresistance of various tumor cells (6–9). However, the upstream mechanism regulating KEAP1/NRF2 signaling is not clear.

Family with sequence similarity 117, member B (FAM117B), protein, containing 589 amino acids, is encoded by a highly conserved gene in animal cells. Previous studies have shown that *FAM117B* is associated with the occurrence of lacunar stroke and sarcoidosis (10, 11). However, the functions of FAM117B in tumors have not been reported. Whether and how FAM117B participates in the progression of gastric cancer cells are not clear. In this study, we found that FAM117B disrupted KEAP1-NRF2 interaction through its ETGE motif, which reduced the degradation of NRF2 and activated KEAP1/NRF2 signaling, and ultimately promoted the growth and chemoresistance of gastric cancer cells. This study may provide a potential therapeutic target for gastric cancer.

## Results

### FAM117B promotes the growth and chemoresistance of gastric cancer cells.

**Figure 1:**
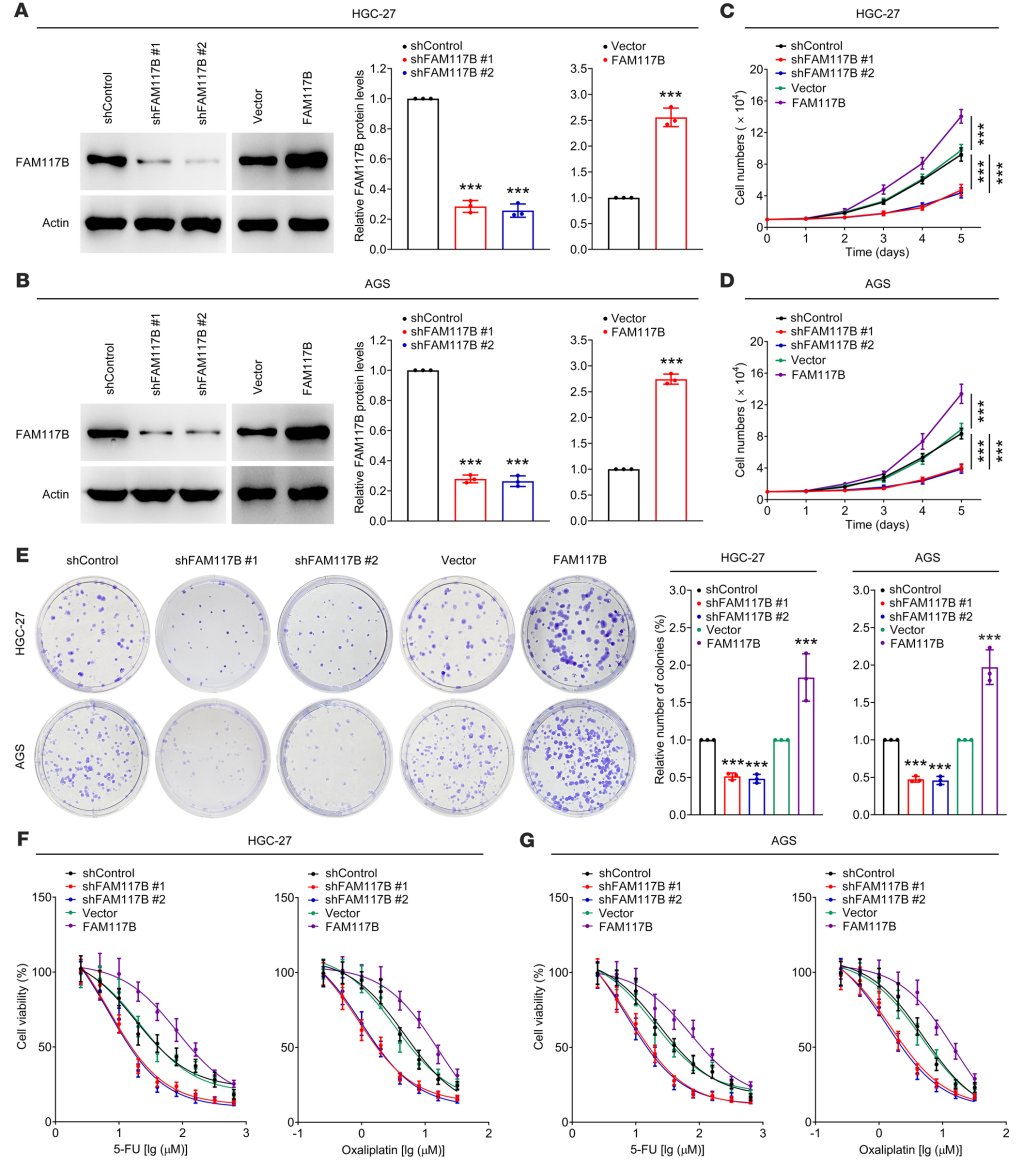
FAM117B promotes the growth and chemoresistance of gastric cancer cells. (**A** and **B**) FAM117B protein levels in HGC-27 and AGS cells transfected with FAM117B shRNAs (#1 and #2) or overexpression plasmids (*n* = 3 independent experiments). (**C** and **D**) Cell growth ability of HGC-27 and AGS cells with FAM117B knockdown or overexpression (*n* = 3 independent experiments). (**E**) Colony formation ability of HGC-27 and AGS cells with FAM117B knockdown or overexpression (*n* = 3 independent experiments). (**F** and **G**) Therapeutic efficacy of 5-FU and oxaliplatin on HGC-27 and AGS cells with FAM117B knockdown or overexpression (*n* = 3 independent experiments). Data are shown as mean ± SD. ***P < 0.001, significant difference vs. shControl or Vector group. Statistical significance was calculated using 2-tailed unpaired Student’s *t* test (2 groups) or 1-way ANOVA followed by Tukey’s test (more than 2 groups).

To study the role of FAM117B in gastric cancer cells, shFAM117B (#1 and #2) and FAM117B overexpression plasmids were used to silence and overexpress FAM117B in HGC-27, AGS, and SNU-668 cells. As shown in Figure 1, A and B, and Supplemental Figure 1A (supplemental material available online with this article; https://doi.org/10.1172/JCI158705DS1), shFAM117B (#1 and #2) and FAM117B overexpression plasmids exhibited strong knockdown and overexpression efficiency, respectively. Subsequently, we investigated the role of FAM117B in regulating the growth and chemoresistance of gastric cancer cells. Figure 1, C–G; Supplemental Figure 1, B–E; and Supplemental Table 1 show that the growth ability, colony formation ability, and chemoresistance of cells with FAM117B knockdown were significantly weakened, while those of FAM117B-overexpressed cells were markedly enhanced. Moreover, restoring FAM117B expression could abolish FAM117B knockdown–induced gastric cancer cell growth inhibition and chemosensitization (Supplemental Figure 2, A–G, and Supplemental Table 2). These data suggest that FAM117B can promote the growth and chemoresistance of gastric cancer cells.

### FAM117B activates KEAP1/NRF2 signaling in gastric cancer cells.

**Figure 2:**
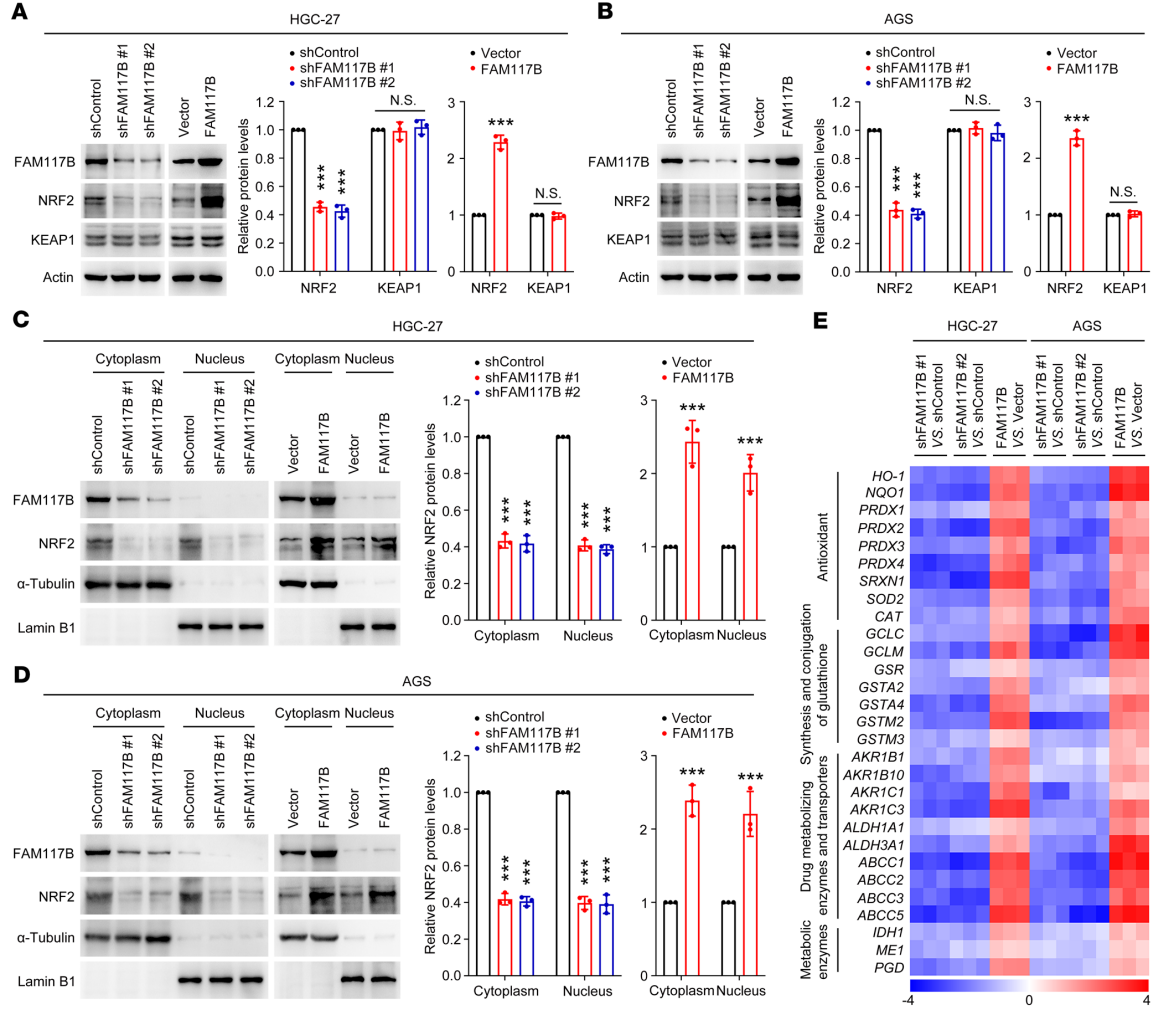
FAM117B activates KEAP1/NRF2 signaling in gastric cancer cells. (**A** and **B**) Protein levels of NRF2 and KEAP1 in HGC-27 and AGS cells with FAM117B knockdown or overexpression (*n* = 3 independent experiments). (**C** and **D**) Protein levels of NRF2 in cytoplasm and nucleus of HGC-27 and AGS cells with FAM117B knockdown or overexpression (*n* = 3 independent experiments). (**E**) mRNA levels of NRF2 target genes in HGC-27 and AGS cells with FAM117B knockdown or overexpression (*n* = 3 independent experiments). The colors of the heatmap represent values of –ΔΔCt. Data are shown as mean ± SD. ***P < 0.001, significant difference vs. shControl or Vector group. Statistical significance was calculated using 2-tailed unpaired Student’s *t* test (2 groups) or 1-way ANOVA followed by Tukey’s test (more than 2 groups).

Previous studies have shown that the KEAP1/NRF2 signaling pathway affects the growth and chemoresistance of gastric cancer cells (12, 13). We then investigated whether FAM117B regulates KEAP1/NRF2 signaling in gastric cancer cells. As shown in Figure 2, A and B; Supplemental Figure 1F; and Supplemental Figure 3, NRF2 protein levels were downregulated when FAM117B was silenced, while upregulated when FAM117B was overexpressed, and in the meantime, the protein levels of KEAP1 remained unchanged (Figure 2, A and B). Restoring FAM117B expression could abolish FAM117B knockdown–induced NRF2 protein inhibition in gastric cancer cells (Supplemental Figure 2, H and I). The nucleoplasmic separation experiment showed that the NRF2 protein levels in both cytoplasm and nucleus were downregulated by FAM117B knockdown and upregulated by FAM117B overexpression (Figure 2, C and D). In addition, we found that the mRNA levels of NRF2 target genes were downregulated and upregulated in cells with FAM117B knockdown and overexpression, respectively (Figure 2E and Supplemental Figure 1G). These data demonstrate that FAM117B activates KEAP1/NRF2 signaling in gastric cancer cells.

### FAM117B activates KEAP1/NRF2 signaling by decreasing ubiquitin-proteasome degradation of NRF2 in gastric cancer cells.

**Figure 3:**
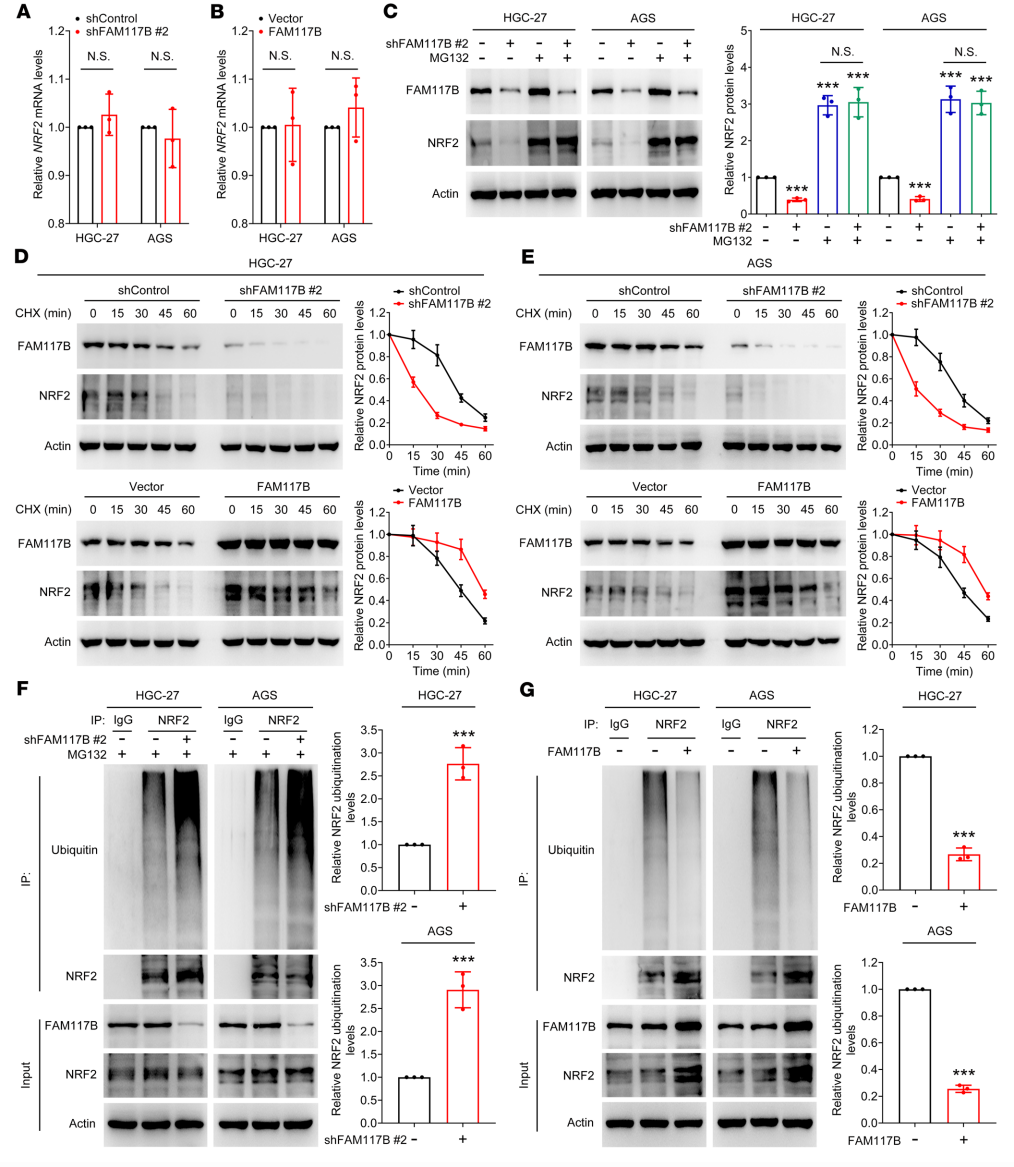
FAM117B activates KEAP1/NRF2 signaling by decreasing ubiquitin-proteasome degradation of NRF2 in gastric cancer cells. (**A** and **B**) mRNA levels of NRF2 in HGC-27 and AGS cells with FAM117B knockdown or overexpression (*n* = 3 independent experiments). (**C**) HGC-27 and AGS cells expressing FAM117B shRNA #2 were treated with or without MG132 (20 μM); Western blot was used to detect the NRF2 protein levels (*n* = 3 independent experiments). (**D** and **E**) NRF2 degradation half-life of HGC-27 and AGS cells with FAM117B knockdown or overexpression (*n* = 3 independent experiments). (**F** and **G**) NRF2 ubiquitination levels of HGC-27 and AGS cells with FAM117B knockdown or overexpression (*n* = 3 independent experiments). Data are shown as mean ± SD. ***P < 0.001, significant difference vs. shControl or Vector group. Statistical significance was calculated using 2-tailed unpaired Student’s *t* test (2 groups) or 1-way ANOVA followed by Tukey’s test (more than 2 groups).

Next, we studied how FAM117B regulates NRF2 protein levels in gastric cancer cells. First, quantitative PCR (qPCR) assay was used to detect mRNA levels of *NRF2* in HGC-27 and AGS cells. As shown in Figure 3, A and B, the changes in FAM117B protein levels did not affect the expression of *NRF2* mRNA, indicating that FAM117B did not elevate the protein level of NRF2 through transcriptional regulation. Therefore, we hypothesized that FAM117B might affect the NRF2 protein level through the degradation pathway. Figure 3C shows that MG132 (a proteasomal inhibitor) deprived shFAM117B #2 of the ability to reduce NRF2 protein levels in cells. The cycloheximide assay showed that knockdown or overexpression of FAM117B shortened or prolonged the half-life of NRF2 protein degradation, respectively (Figure 3, D and E). We also examined the effect of FAM117B on NRF2 ubiquitination in HGC-27 and AGS cells; as shown in Figure 3, F and G, FAM117B knockdown markedly upregulated the ubiquitination levels of NRF2, and FAM117B overexpression significantly downregulated it. These data indicate that FAM117B activates KEAP1/NRF2 signaling by decreasing ubiquitin-proteasome degradation of NRF2 in gastric cancer cells.

### FAM117B competes with NRF2 for KEAP1 binding in gastric cancer cells.

**Figure 4:**
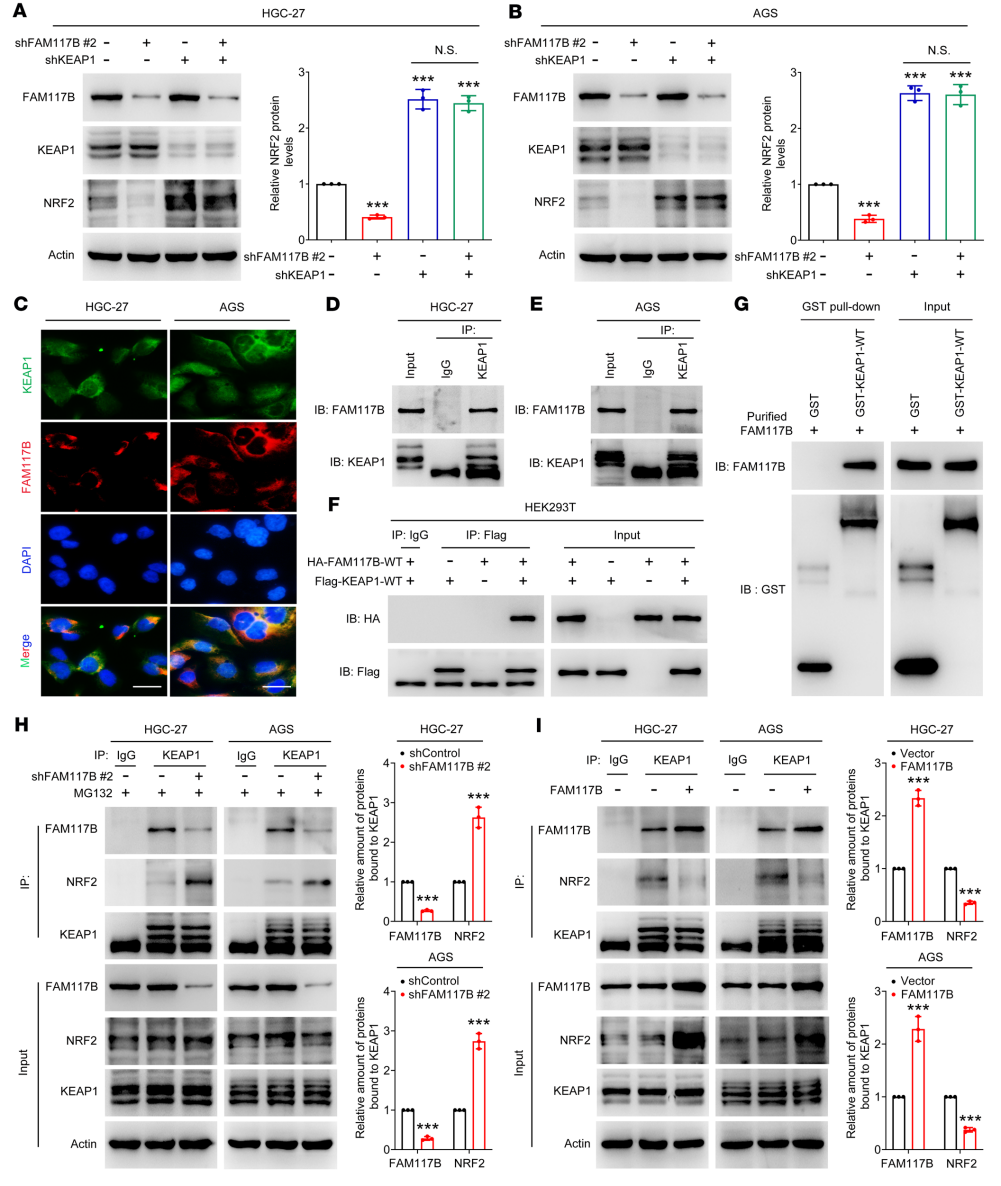
FAM117B competes with NRF2 for KEAP1 binding in gastric cancer cells. (**A** and **B**) NRF2 protein levels in HGC-27 and AGS cells expressing indicated shRNAs (*n* = 3 independent experiments). (**C**) Immunofluorescence assay (*n* = 3 independent experiments). Scale bars: 10 μm. (**D** and **E**) Binding of endogenous FAM117B to KEAP1 in HGC-27 and AGS cells (*n* = 3 independent experiments). (**F**) Binding of exogenous HA-FAM117B-WT to FLAG-KEAP1-WT in HEK293T cells (*n* = 3 independent experiments). (**G**) Binding of purified GST-KEAP1-WT to purified FAM117B (*n* = 3 independent experiments). (**H** and **I**) Binding levels of FAM117B or NRF2 to KEAP1 in HGC-27 and AGS cells with FAM117B knockdown or overexpression (*n* = 3 independent experiments). ***P < 0.001, significant difference vs. shControl or Vector group. Statistical significance was calculated using 2-tailed unpaired Student’s *t* test (2 groups) or 1-way ANOVA followed by Tukey’s test (more than 2 groups).

Given that the ubiquitination degradation of NRF2 is regulated by KEAP1, we surmised that FAM117B-induced upregulation of NRF2 was dependent on KEAP1. As shown in Figure 4, A and B, shFAM117B #2 lost its ability to downregulate NRF2 protein levels in KEAP1-silenced cells, suggesting that FAM117B regulates NRF2 in a KEAP1-dependent manner. The immunofluorescence assay showed that FAM117B and KEAP1 were colocalized in the cytoplasm of HGC-27 and AGS cells (Figure 4C). The immunoprecipitation assay showed that endogenous FAM117B protein was able to interact with endogenous KEAP1 protein (Figure 4, D and E). Moreover, HA-tagged FAM117B interacted with FLAG-tagged KEAP1 in HEK293T cells (Figure 4F). To demonstrate whether FAM117B is directly bound to KEAP1, the GST pull-down assay was performed. As shown in Figure 4G, GST-KEAP1 protein, but not GST protein, could pull down purified FAM117B protein, suggesting that FAM117B directly binds to KEAP1. Subsequently, we investigated whether FAM117B could impede the binding of NRF2 to KEAP1 in HGC-27 and AGS cells. Figure 4, H and I, shows that FAM117B knockdown or overexpression promoted or inhibited the binding of KEAP1 to NRF2, respectively. We then asked why FAM117B could compete with NRF2 to bind KEAP1. To this end, the microscale thermophoresis assay was used to detect the affinity between FAM117B and KEAP1 and the affinity between NRF2 and KEAP1. As shown in Supplemental Figure 4, A and B, the affinity between NRF2 and KEAP1 (*K_D_* = 4.69 ± 0.19 μM) was stronger than that between FAM117B and KEAP1 (*K_D_* = 7.97 ± 0.43 μM). Considering that NRF2 has 2 motifs (ETGE and DLG) that can bind KEAP1, we truncated the DLG motif of NRF2, then detected the affinity between truncated NRF2 (NRF2^ΔDLG^) and KEAP1. However, the affinity between NRF2^ΔDLG^ and KEAP1 (*K_D_* = 5.28 ± 0.37 μM) was still stronger than that between FAM117B and KEAP1 (Supplemental Figure 4C). These results did not seem to explain why FAM117B competed with NRF2 for KEAP1 binding. Subsequently, we analyzed the protein levels of FAM117B and NRF2 in gastric cancer tumor tissues and gastric cancer cells and found that the protein levels of FAM117B in gastric cancer tumor tissues and gastric cancer cells were significantly higher than that of NRF2 (Supplemental Figure 4, D–F). We hypothesized that the higher protein level of FAM117B in gastric cancer could compensate for the lower affinity between FAM117B and KEAP1. These results reveal that FAM117B competes with NRF2 for KEAP1 binding in gastric cancer cells.

### FAM117B binds to the DGR domain of KEAP1 through its ETGE motif.

**Figure 5:**
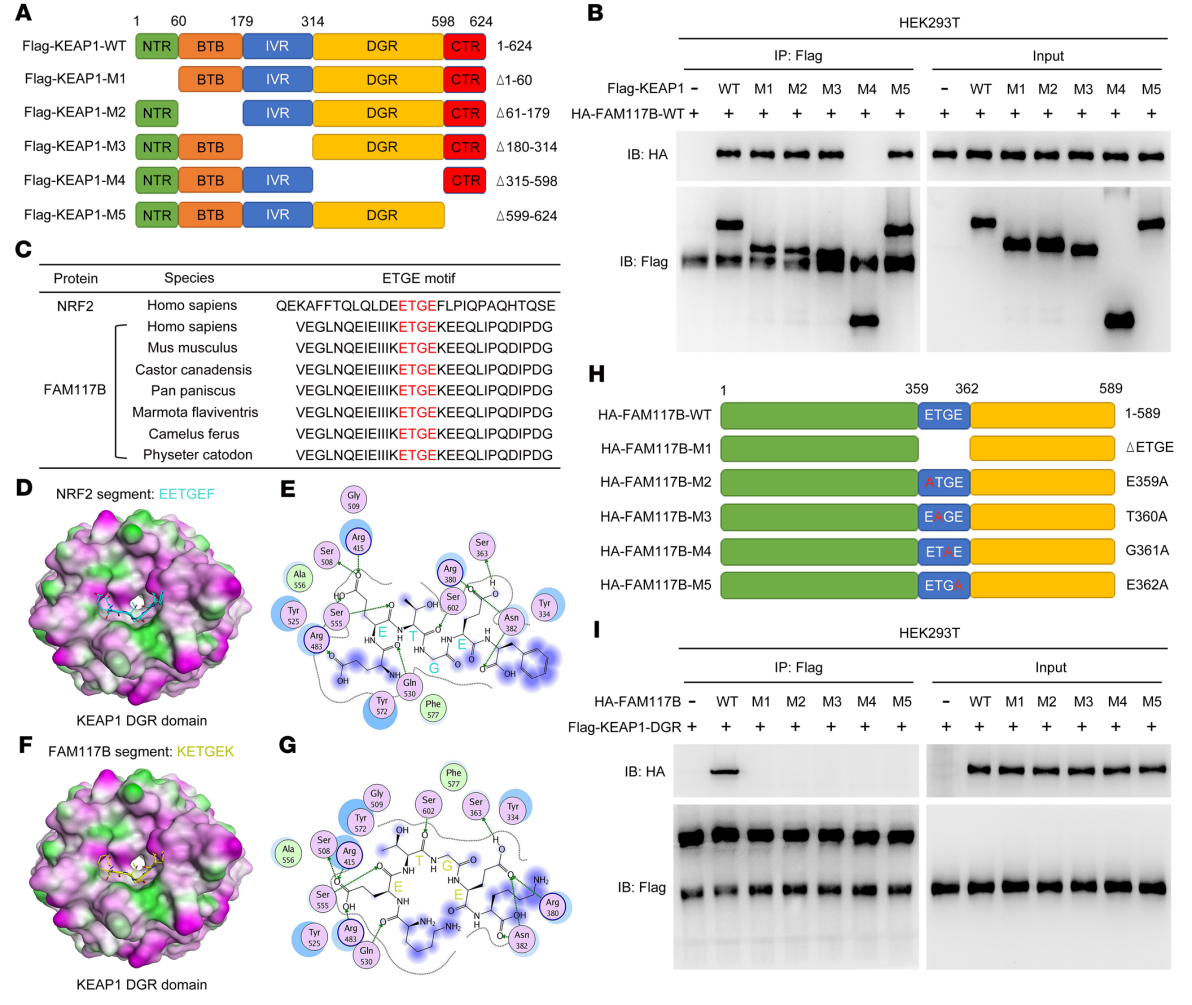
FAM117B binds to the DGR domain of KEAP1 through its ETGE motif. (**A**) Diagram of WT and truncated mutants of KEAP1. (**B**) Binding of exogenous HA-FAM117B-WT to FLAG-KEAP1-WT or truncated mutants in HEK293T cells (*n* = 3 independent experiments). (**C**) ETGE motif of FAM117B in different species. (**D**–**G**) Interaction of the ETGE motifs of NRF2 and FAM117B with crystal structure of KEAP1-DGR. (**H**) Diagram of WT and mutants of FAM117B. (**I**) Binding of exogenous FLAG-KEAP1-DGR to HA-FAM117B-WT or mutants in HEK293T cells (*n* = 3 independent experiments).

Previous studies have shown that KEAP1 contains 5 functional domains: the NTR (amino acids 1–60), BTB (amino acids 61–179), IVR (amino acids 180–314), DGR (amino acids 315–598), and CTR (amino acids 599–624) domains (14). To determine which domains of KEAP1 bind to FAM117B, FLAG-tagged KEAP1-WT and functional domain–truncated mutant plasmids (Figure 5A) were constructed and cotransfected into HEK293T cells with HA-tagged FAM117B-WT (HA-FAM117B-WT) plasmids, and then the immunoprecipitation assay was performed. As shown in Figure 5B, HA-FAM117B-WT protein could not bind to FLAG-KEAP1-M4 mutant protein (DGR domain deletion), indicating that the DGR domain of KEAP1 is essential for its interaction with FAM117B.

In order to study the functional motifs of FAM117B that bind with the DGR domain of KEAP1, the amino acid sequences of FAM117B proteins from different species were analyzed. Figure 5C shows that the FAM117B proteins of these different species contained the ETGE motif. Since NRF2 binds to the DGR domain of KEAP1 mainly through its ETGE motif, we hypothesized that the ETGE motif of FAM117B protein might have similar function. To test this hypothesis, the bindings of NRF2 peptide containing the ETGE motif and FAM117B peptide containing the same motif to the DGR domain of KEAP1 were analyzed by molecular docking. As shown in Figure 5, D–G, both NRF2 and FAM117B peptides containing the ETGE motif bound to the same active site in the DGR domain of KEAP1, and interacted with key amino acids (Ser508, Arg415, Arg483, Ser555, Ser602, Arg380, Ser363, Asn382) within the active site, suggesting that the ETGE motif of FAM117B may be an essential motif for binding to KEAP1. To verify this, we constructed HA-tagged FAM117B-WT and different mutant plasmids (Figure 5H), then cotransfected these plasmids into HEK293T cells with FLAG-tagged KEAP1 DGR (FLAG-KEAP1-DGR) plasmids. Figure 5I shows that HA-FAM117B-M1 (ETGE motif deletion), HA-FAM117B-M2 (E359A), HA-FAM117B-M3 (T360A), HA-FAM117B-M4 (G361A), and HA-FAM117B-M5 (E362A) proteins could not bind to FLAG-KEAP1-DGR protein, indicating that the ETGE motif of FAM117B is essential for its binding to the DGR domain of KEAP1. These results demonstrate that FAM117B binds to the DGR domain of KEAP1 through its ETGE motif.

### The ETGE motif of FAM117B activates KEAP1/NRF2 signaling and promotes the growth and chemoresistance of gastric cancer cells.

**Figure 6:**
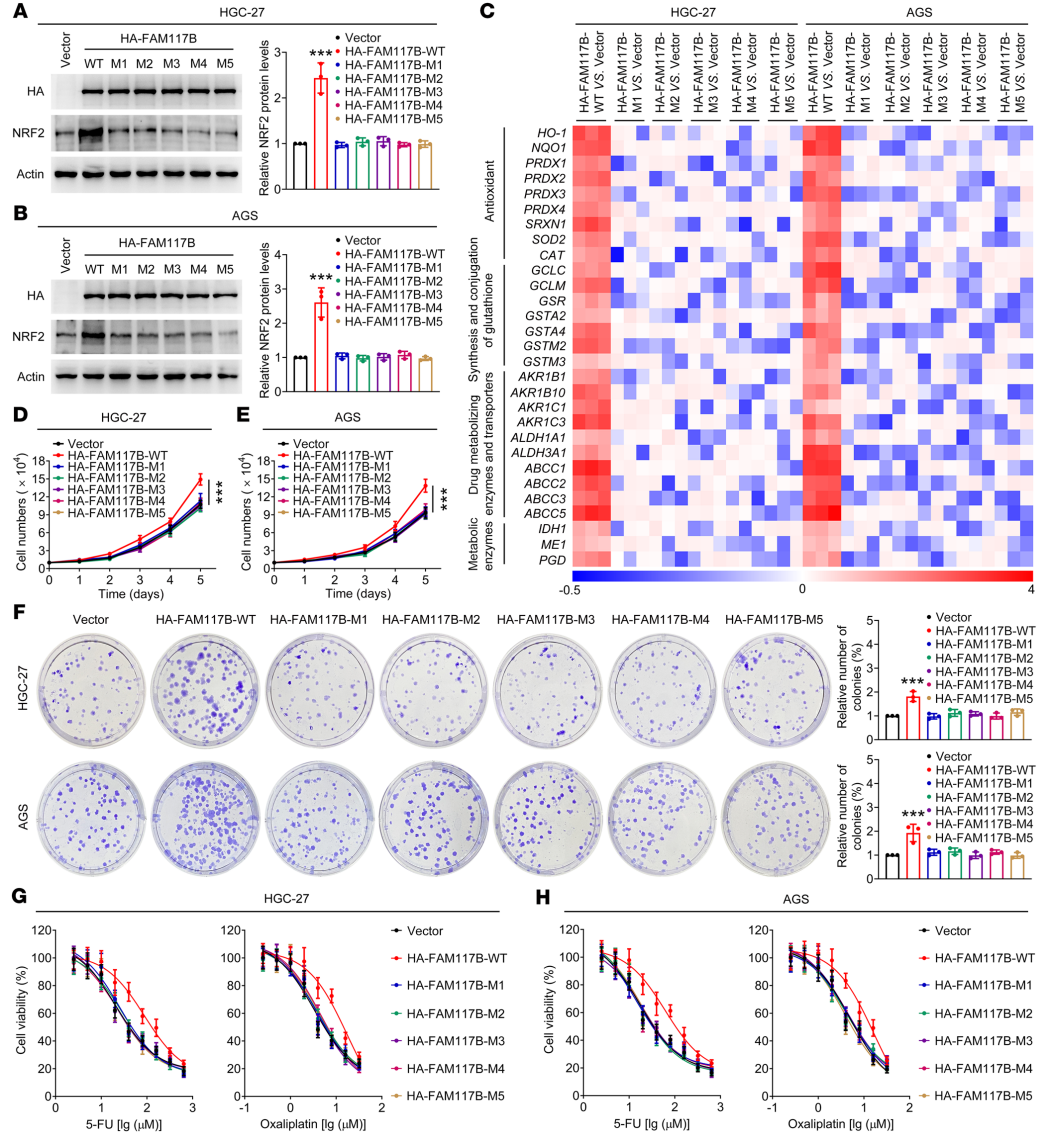
The ETGE motif of FAM117B activates KEAP1/NRF2 signaling and promotes the growth and chemoresistance of gastric cancer cells. (**A** and **B**) Protein levels of NRF2 in HGC-27 and AGS cells transfected with HA-FAM117B-WT or mutant plasmids (*n* = 3 independent experiments). (**C**) mRNA levels of NRF2 target genes in HGC-27 and AGS cells transfected with HA-FAM117B-WT or mutant plasmids (*n* = 3 independent experiments). The colors of the heatmap represent values of –ΔΔCt. (**D** and **E**) Cell growth ability of HGC-27 and AGS cells transfected with HA-FAM117B-WT or mutant plasmids (*n* = 3 independent experiments). (**F**) Colony formation ability of HGC-27 and AGS cells transfected with HA-FAM117B-WT or mutant plasmids (*n* = 3 independent experiments). (**G** and **H**) Therapeutic efficacy of 5-FU and oxaliplatin to HGC-27 and AGS cells transfected with HA-FAM117B-WT or mutant plasmids (*n* = 3 independent experiments). ***P < 0.001, significant difference vs. Vector group. Statistical significance was calculated using 1-way ANOVA followed by Tukey’s test.

To study the effect of the ETGE motif of FAM117B on regulation of KEAP1/NRF2 signaling in gastric cancer cells, HA-tagged FAM117B WT and ETGE mutants were expressed in HGC-27 and AGS cells, and then the protein levels of NRF2 and the mRNA levels of its downstream target genes were detected by Western blot and qPCR assays. As shown in Figure 6, A–C, HA-FAM117B-WT could upregulate the protein levels of NRF2 and the mRNA levels of its downstream target genes, while the HA-FAM117B-ETGE mutants did not have the same effect, suggesting that the ETGE motif of FAM117B is essential for its regulation of KEAP1/NRF2 signaling. Subsequently, we investigated the role of the ETGE motif of FAM117B in regulating the growth and chemoresistance of gastric cancer cells. As shown in Figure 6, D–H, and Supplemental Table 3, HA-FAM117B-WT was able to promote the growth, colony formation ability, and chemoresistance of HGC-27 and AGS cells, while HA-FAM117B-ETGE mutants lost these functions. In addition, we found that the ETGE motif of FAM117B was also able to promote the growth and chemoresistance of HGC-27 cell–derived xenografts (Supplemental Figures 5 and 6). These data indicate that FAM117B can activate KEAP1/NRF2 signaling and promote the growth and chemoresistance of gastric cancer cells through its ETGE motif.

### FAM117B promotes the growth of gastric cancer cells via regulation of KEAP1/NRF2 signaling.

**Figure 7:**
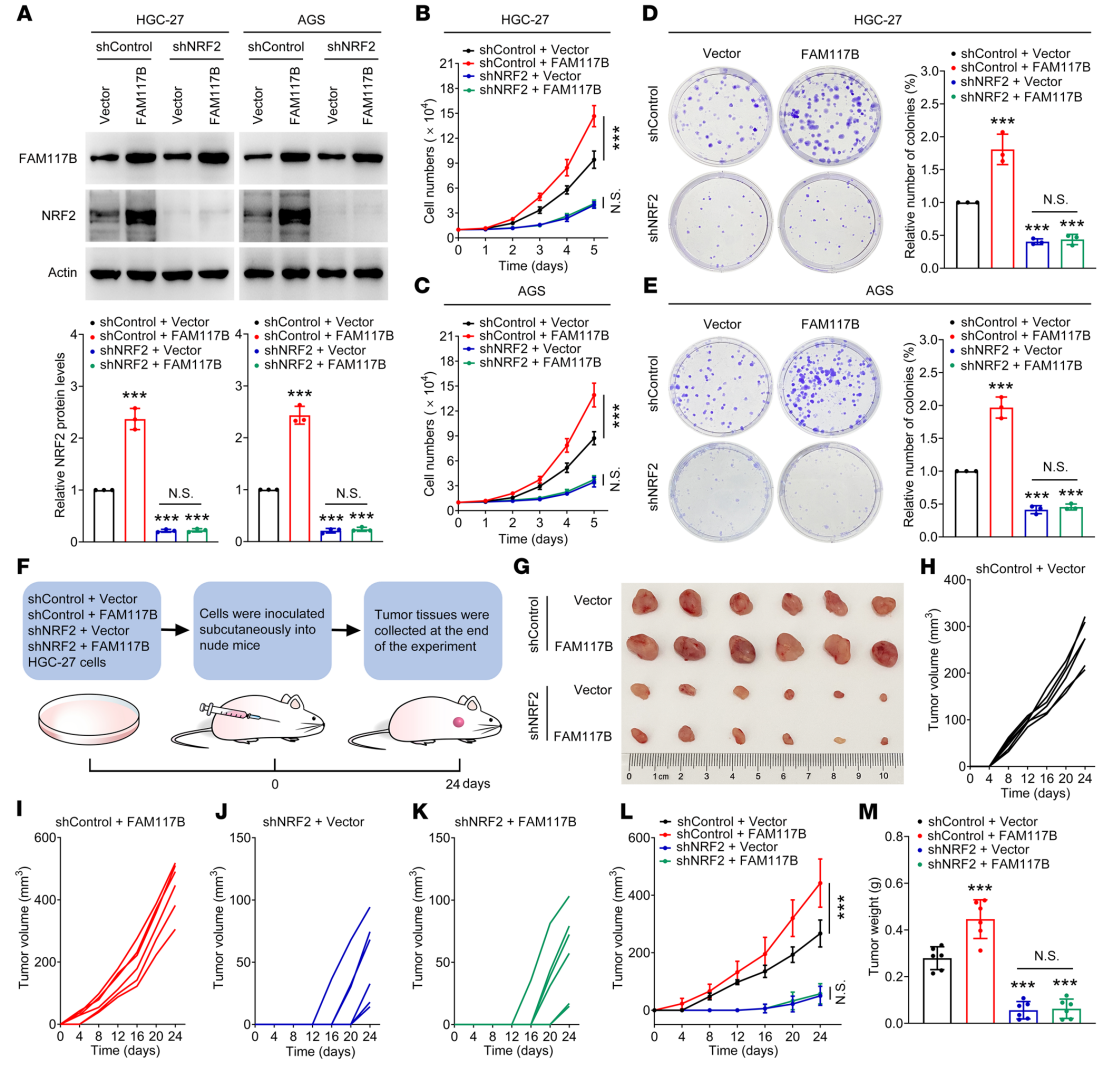
FAM117B promotes the growth of gastric cancer cells via regulation of KEAP1/NRF2 signaling. (**A**) Protein levels of NRF2 in HGC-27 and AGS cells transfected with shControl + Vector, shControl + FAM117B, shNRF2 + Vector, and shNRF2 + FAM117B (*n* = 3 independent experiments). (**B** and **C**) Cell growth ability of HGC-27 and AGS cells transfected with shControl + Vector, shControl + FAM117B, shNRF2 + Vector, and shNRF2 + FAM117B (*n* = 3 independent experiments). (**D** and **E**) Colony formation ability of HGC-27 and AGS cells transfected with shControl + Vector, shControl + FAM117B, shNRF2 + Vector, and shNRF2 + FAM117B (*n* = 3 independent experiments). (**F**) Schematic diagram of the in vivo study. (**G**) Image of tumors (*n* = 6 per group). (**H**–**L**) Growth curves of tumor volume (*n* = 6 per group). (**M**) Tumor weight (*n* = 6 per group). ***P < 0.001, significant difference vs. shControl + Vector group. Statistical significance was calculated using 1-way ANOVA followed by Tukey’s test.

To investigate whether FAM117B-induced growth of gastric cancer cells was NRF2 dependent, FAM117B was overexpressed in NRF2-silenced gastric cancer cells, and then the abilities of cell growth and colony formation were detected. As shown in Figure 7A and Supplemental Figure 7A, FAM117B significantly upregulated the protein levels of NRF2 in shControl–gastric cancer cells, while having no marked effect on the protein levels of NRF2 in NRF2-silenced cells. Moreover, the abilities of cell growth and colony formation in shControl–gastric cancer cells with FAM117B overexpression were significantly enhanced, while overexpression of FAM117B did not show similar effects in NRF2-silenced cells (Figure 7, B–E, and Supplemental Figure 7, B and C). We also found that FAM117B overexpression downregulated ROS levels in shControl–gastric cancer cells, but not in NRF2-silenced cells (Supplemental Figure 8), indicating that FAM117B induces NRF2 to regulate ROS levels in gastric cancer cells. Subsequently, we constructed the shControl + Vector, shControl + FAM117B, shNRF2 + Vector, and shNRF2 + FAM117B–HGC-27 and SNU-668 cell–derived xenografts and observed their growth (Figure 7F and Supplemental Figure 7D). As shown in Figure 7, G–M, and Supplemental Figure 7, E–K, FAM117B significantly promoted the growth of shControl–HGC-27 and SNU-668 cell–derived xenografts but did not have this effect on shNRF2–HGC-27 and SNU-668 cell–derived xenografts. Moreover, the immunohistochemical (IHC) experiment showed that FAM117B markedly promoted the protein levels of NRF2 and positive rates of Ki67 in shControl–HGC-27 cell–derived xenografts but had no effect on these 2 proteins in shNRF2–HGC-27 cell–derived xenografts (Supplemental Figure 9). In addition, we evaluated the angiogenesis and cell apoptosis of these xenografts. As shown in Supplemental Figure 10, FAM117B could significantly promote angiogenesis and inhibit cell apoptosis in shControl–HGC-27 cell–derived xenografts, while it lost the ability to promote angiogenesis and inhibit cell apoptosis in shNRF2–HGC-27 cell–derived xenografts, indicating that FAM117B-induced angiogenesis promotion and cell apoptosis inhibition effects rely on NRF2. These data suggest that FAM117B-induced growth of gastric cancer cells is NRF2 dependent.

### FAM117B promotes the chemoresistance of gastric cancer cells via regulation of KEAP1/NRF2 signaling.

**Figure 8:**
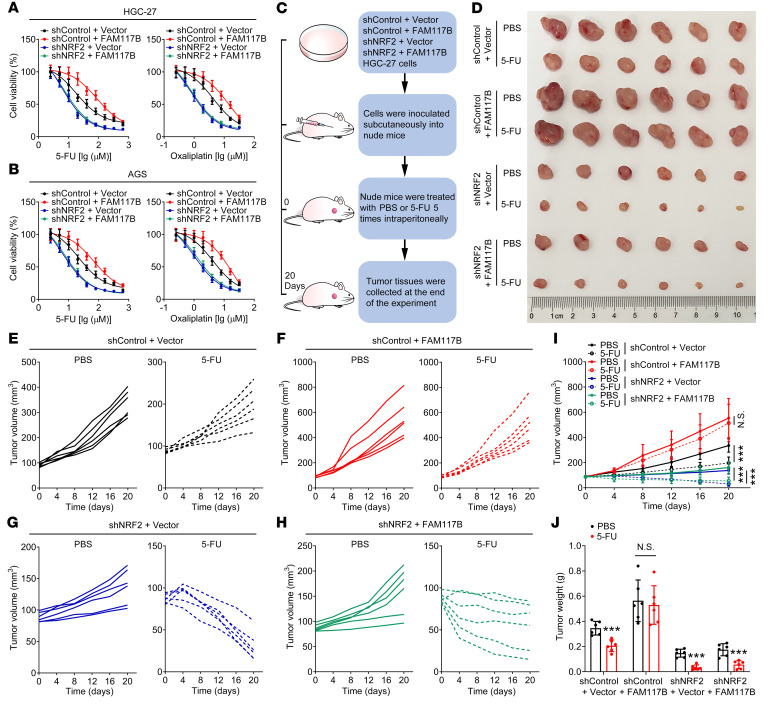
FAM117B promotes the chemoresistance of gastric cancer cells via regulation of KEAP1/NRF2 signaling. (**A** and **B**) Therapeutic efficacy of 5-FU and oxaliplatin to HGC-27 and AGS cells transfected with shControl + Vector, shControl + FAM117B, shNRF2 + Vector, and shNRF2 + FAM117B (*n* = 3 independent experiments). (**C**) Schematic diagram of the in vivo study. (**D**) Image of tumors (*n* = 6 per group). (**E**–**I**) Growth curves of tumor volume (*n* = 6 per group). (**J**) Tumor weight (*n* = 6 per group). Data are shown as mean ± SD. ***P < 0.001, significant difference vs. PBS group. Statistical significance was calculated using 1-way ANOVA followed by Tukey’s test.

To study whether FAM117B-induced chemoresistance of gastric cancer cells was NRF2 dependent, MTT assay was performed. As shown in Figure 8, A and B; Supplemental Figure 11, A and B; and Supplemental Table 4, after overexpression of FAM117B in shControl–gastric cancer cells, the sensitivities of cells to fluorouracil (5-FU) and oxaliplatin were markedly reduced, while FAM117B overexpression did not significantly diminish the sensitivities of shNRF2–gastric cancer cells to these 2 drugs. Subsequently, the shControl + Vector, shControl + FAM117B, shNRF2 + Vector, and shNRF2 + FAM117B–HGC-27 and SNU-668 cell–derived xenograft models were constructed to further validate that FAM117B-induced chemoresistance was NRF2 dependent (Figure 8C and Supplemental Figure 11C). The results of in vivo experiments showed that 5-FU had a marked inhibitory effect on the growth of xenografts in the shControl + Vector group but had no similar inhibitory effect in the shControl + FAM117B group (Figure 8, D–J, and Supplemental Figure 11, D–J). Meanwhile, as with the shNRF2 + Vector group, 5-FU still exhibited a strong inhibitory effect on the growth of xenografts in the shNRF2 + FAM117B group (Figure 8, D–J, and Supplemental Figure 11, D–J). In addition, we found that 5-FU significantly inhibited the positive rates of Ki67 in xenograft tissues of the shControl + Vector, shNRF2 + Vector, and shNRF2 + FAM117B groups but had no effect on Ki67 in xenograft tissues of the shControl + FAM117B group (Supplemental Figure 12). These data indicate that FAM117B-induced chemoresistance of gastric cancer cells is NRF2 dependent.

### FAM117B and NRF2 are both overexpressed in gastric cancer tissues, and their co-overexpression represents a factor for poor prognosis.

The protein levels of FAM117B and NRF2 in tumor tissues and matched adjacent normal tissues of gastric cancer patients were detected by Western blot. As shown in Supplemental Figure 13, A–C, the protein levels of FAM117B and NRF2 in gastric cancer tumor tissues were significantly higher than those in adjacent normal tissues. Subsequently, tumor samples were divided into 2 groups, low FAM117B protein level and high FAM117B protein level, and NRF2 protein levels in these 2 groups were analyzed. Supplemental Figure 13D shows that the protein level of NRF2 in the group with a high FAM117B protein level was significantly higher than that in the group with a low FAM117B protein level. The Spearman correlation analysis demonstrated a strong correlation between the protein levels of FAM117B and NRF2 in gastric cancer tissues (Supplemental Figure 13E).

**Figure 9:**
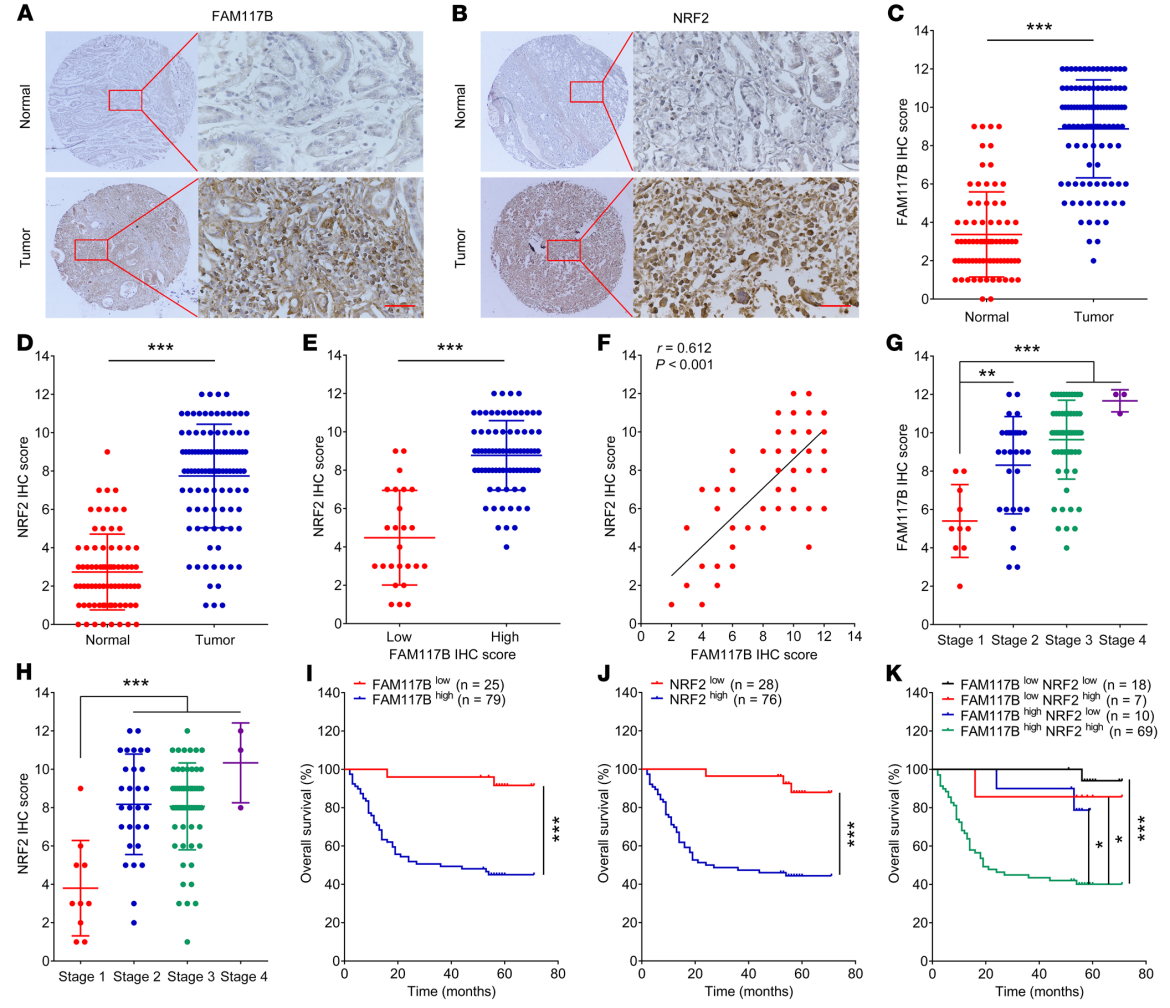
FAM117B and NRF2 are both overexpressed in gastric cancer tissues, and their co-overexpression represents a factor for poor prognosis. (**A**–**D**) FAM117B and NRF2 IHC scores of tumor tissues (*n* = 104) and adjacent normal tissues (*n* = 76) in SOB cohort. Scale bars: 50 μm. (**E**) IHC scores of NRF2 in tumor tissues with low (*n* = 25) or high (*n* = 79) FAM117B IHC score. (**F**) Spearman correlation analysis of FAM117B and NRF2 IHC scores in tumor tissues (*n* = 104). (**G** and **H**) IHC scores of FAM117B and NRF2 in tumor tissues with clinical stage 1 (*n* = 10), stage 2 (*n* = 29), stage 3 (*n* = 61), and stage 4 (*n* = 3). One patient’s clinical stage information was missing. (**I**–**K**) Kaplan-Meier survival analysis. Data are shown as mean ± SD. *P < 0.05, **P < 0.01, and ***P < 0.001 indicate significant difference. Statistical significance was calculated using 2-tailed unpaired Student’s *t* test (2 groups), 1-way ANOVA followed by Tukey’s test (more than 2 groups), 2-tailed Spearman test, or log-rank test.

Next, the IHC assay was used to detect the protein levels of FAM117B and NRF2 of tumor tissue and adjacent normal tissues in gastric cancer microarrays (SOB cohort and AFB cohort). As shown in Figure 9, A–D, and Supplemental Figure 14, A–D, the IHC scores of FAM117B and NRF2 in gastric cancer tumor tissues were significantly higher than those in adjacent normal tissues. Subsequently, we divided the tumor tissues into a high IHC score group and a low IHC score group, then analyzed the IHC scores of NRF2 in these 2 groups. As shown in Figure 9E and Supplemental Figure 14E, the NRF2 IHC score of the group with a high FAM117B score was significantly higher than that of the group with a low FAM117B score. The Spearman correlation analysis indicated a significant correlation between the IHC scores of FAM117B and NRF2 in gastric cancer tissues (Figure 9F and Supplemental Figure 14F). Moreover, we also analyzed the IHC scores of FAM117B and NRF2 of gastric cancer tumor tissues in various clinical stages. As shown in Figure 9, G and H, and Supplemental Figure 14, G and H, the IHC scores of FAM117B and NRF2 in stages 2–4 were significantly higher than those in stage 1, indicating that FAM117B and NRF2 protein levels positively correlate with clinical stage.

The Kaplan-Meier survival analysis showed that patients with high FAM117B IHC scores had a markedly shorter overall survival than those with low FAM117B IHC scores (Figure 9I and Supplemental Figure 14I). Moreover, patients with high NRF2 IHC scores also had significantly shorter overall survival (Figure 9J and Supplemental Figure 14J). We then divided the patients into 4 groups: low FAM117B/low NRF2 IHC scores; low FAM117B/high NRF2 IHC scores; high FAM117B/low NRF2 IHC scores; and high FAM117B/high NRF2 IHC scores. As shown in Figure 9K and Supplemental Figure 14K, patients with high FAM117B and high NRF2 IHC scores in tumor tissues had the shortest overall survival. The univariate Cox regression analysis and multivariate Cox regression analysis revealed that patients with high FAM117B and high NRF2 IHC scores had a markedly high risk of death (Supplemental Table 5). These data demonstrate that FAM117B and NRF2 are both overexpressed in gastric cancer tissues, and their co-overexpression represents an independent factor for poor prognosis.

## Discussion

**Figure 10:**
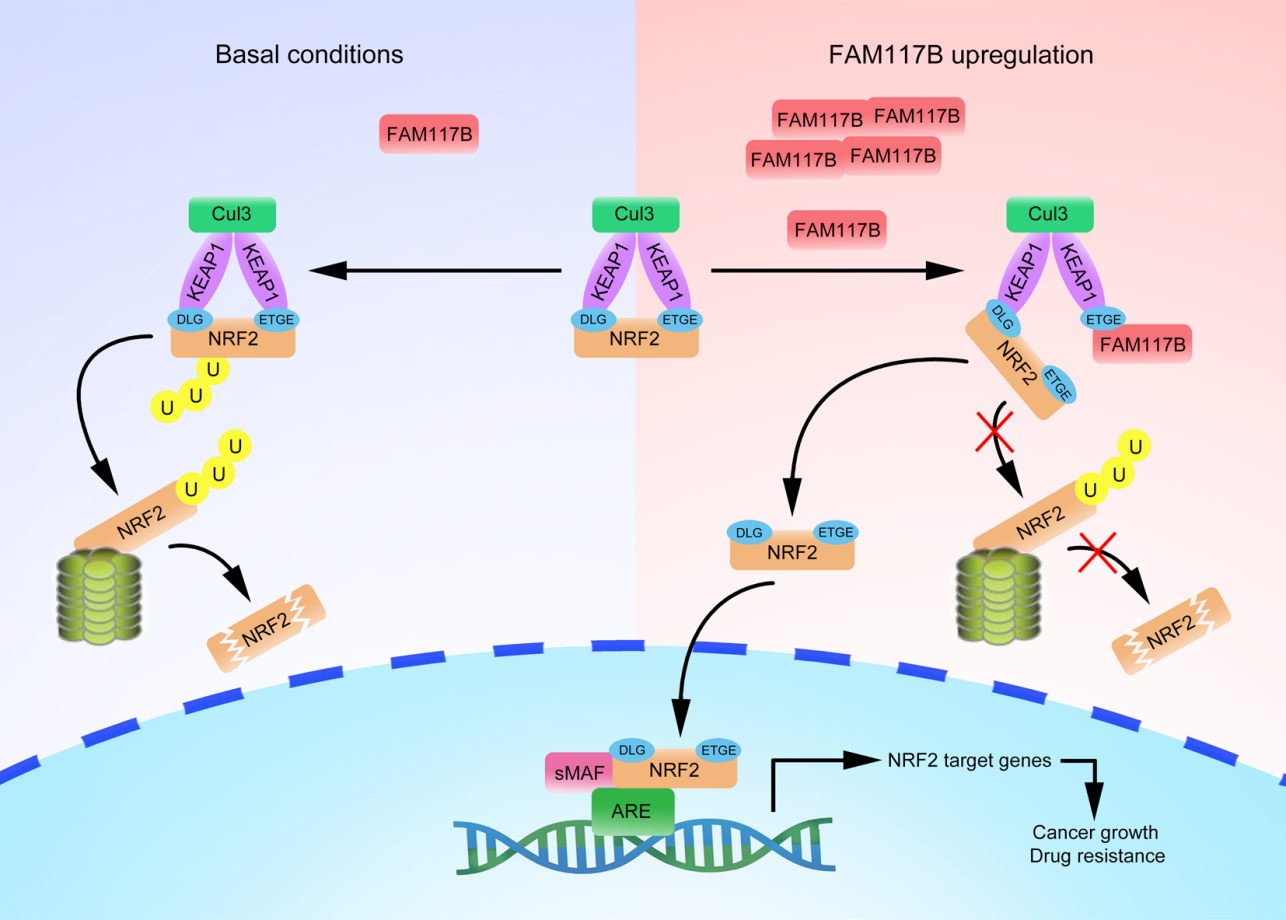
Schematic diagram of FAM117B regulating KEAP1/NRF2 signaling in gastric cancer cells. Upregulation of FAM1117B disrupts KEAP1-NRF2 interaction, which reduces the ubiquitination degradation of NRF2 and activates KEAP1/NRF2 signaling, and ultimately promotes the growth and chemoresistance of gastric cancer cells.

Excessive activation of abnormal signals can lead to the malignant growth and chemoresistance of gastric cancer, hindering its effective treatment. Despite growing research efforts, the exact molecular mechanism remains unclear. Here, we report that FAM117B competitively inhibited the binding of NRF2 and KEAP1 and activated the KEAP1/NRF2 signaling pathway by decreasing the ubiquitination degradation of NRF2, which in turn promoted the growth of gastric cancer cells and reduced the sensitivity of cells to chemotherapeutic drugs (Figure 10). Our studies reveal the mechanism by which FAM117B promotes the growth and chemoresistance of gastric cancer and may provide a novel therapeutic target for gastric cancer.

As one of the major antioxidant response signaling pathways, the KEAP1/NRF2 system is closely associated with tumor development and chemoresistance. In the canonical KEAP1/NRF2 system, NRF2 binds to KEAP1 homodimers through its ETGE (higher affinity) and DLG (lower affinity) motifs (15). This binding mode promotes the ubiquitination degradation of NRF2. Recently, one study has shown that the binding between KEAP1 and NRF2 is dynamic (16). In cells, “open” conformation (NRF2 binds to KEAP1 through the DLG motif only) coexists with “closed” conformation (NRF2 binds to KEAP1 through both the DLG and the ETGE motif). NRF2 is ubiquitinated and then degraded by the proteasome when the conformation of the KEAP1/NRF2 complex is converted from “open” to “closed.” Since FAM117B can inhibit the ubiquitination degradation of NRF2, it may facilitate the conformation change of the KEAP1/NRF2 complex from “closed” to “open.” Although the amino acid sequence of FAM117B contains an ETGE motif, we found that the affinity of FAM117B to KEAP1 was weaker than that of NRF2^ΔDLG^ to KEAP1. It seems untenable that FAM117B and NRF2 competitively bind with KEAP1 to change the conformation of the KEAP1/NRF2 complex. Yet we found that the protein level of FAM117B in gastric cancer was higher than that of NRF2. The higher protein level of FAM117B in gastric cancer could compensate for the lower affinity between FAM117B and KEAP1. Therefore, it was not surprising that FAM117B competed with NRF2 to bind KEAP1 in gastric cancer cells, changed the conformation of the KEAP1/NRF2 complex, and inhibited the ubiquitination degradation of NRF2. In addition, recent studies have reported that some proteins containing the ETGE motif (e.g., CDK20, WTX, DPP3, and PALB2) regulate KEAP1/NRF2 signaling by binding to KEAP1 (17–20), implying that the ETGE motif plays an important role in binding to KEAP1. Indeed, our data also demonstrated the importance of the ETGE motif of FAM117B in binding to KEAP1 and activating KEAP1/NRF2 signaling. Based on previous studies and our own results, we surmised that the ETGE motif–containing proteins might potentially bind to KEAP1 and regulate KEAP1/NRF2 signaling in cancer cells. However, more studies are needed to confirm this hypothesis. Actually, the overactivation of the KEAP/NRF2 signaling pathway is directly regulated not only by these KEAP1-binding proteins but also by other factors. Some studies indicated that the mutations in the *NRF2* and *KEAP1* genes occur frequently in tumors and drive overactivation of KEAP1/NRF2 signaling (21–25). In addition to the mutations in *NRF2* and *KEAP1* genes, *KRAS* mutations can also activate KEAP1/NRF2 signaling through complex signaling transduction mechanisms (26–29). Considering that KEAP1/NRF2 signaling is regulated by multiple mechanisms in tumor cells, further studies are needed to determine the importance of FAM117B-mediated regulation of KEAP1/NRF2 signaling in gastric cancer.

Conventional chemotherapeutic drugs for gastric cancer (e.g., 5-FU, platinum, taxanes, and anthracyclines) are currently facing a bottleneck in efficacy. The overactivation of the KEAP1/NRF2 signaling pathway in tumor cells is one important reason for the poor efficacy of these drugs. In recent years, with the establishment of immunotherapy in the first-line treatment of advanced gastric cancer, the treatment of gastric cancer has undergone earth-shaking changes. Among all immunotherapies, anti–programmed cell death 1 (PD-1)/programmed cell death ligand 1 (PD-L1) immune checkpoint inhibitors are the most widely used. Singh et al. reported that tumors with NRF2 activation are not sensitive to anti–PD-L1 treatment (30). Furthermore, Cristescu et al. reported that tumors with *KEAP1* mutation are immunologically “cold” and do not respond well to immune checkpoint blockade (31). Given the reason NRF2 is overactivated in gastric cancer, NRF2 could be considered a potential drug target. Since NRF2 is a member of the basic leucine zipper transcription factor family involved in the regulation of diverse and critical biological functions (9, 32), transcriptional inhibitors of NRF2 may bind other basic leucine zipper transcription factors and cause some adverse side effects. This is one reason researchers have not successfully developed specific transcriptional inhibitors of NRF2. Therefore, exploring the upstream regulation mechanism of KEAP1/NRF2 signaling via protein-protein interactions may provide an alternative for the inhibition of NRF2. Our findings revealed that FAM117B could bind KEAP1 to upregulate NRF2, then attenuated the sensitivity of gastric cancer to chemotherapeutic drugs. Therefore, the development of small molecules that specifically bind FAM117B and interfere with the FAM117B/KEAP1 complex may effectively inhibit NRF2 without causing adverse side effects.

Notably, effective prognostic factors for gastric cancer are still scarce in the clinic and need to be discovered. Our study showed that FAM117B protein levels were elevated in human gastric cancer samples. Moreover, FAM117B overexpression was significantly associated with a poor prognosis of patients with gastric cancer. In addition, we demonstrated that high expression of NRF2 in gastric cancer tissues was significantly associated with a poor prognosis of patients (Figure 9). More importantly, patients with high protein levels of both FAM117B and NRF2 had worse prognosis than patients in the other groups. These results suggest that FAM117B and NRF2 protein levels in tumor tissues are reliable prognostic factors. Given the above findings that FAM117B activates KEAP1/NRF2 signaling and promotes chemoresistance, the combination of small-molecule inhibitors that specifically bind FAM117B and interfere with the FAM117B/KEAP1 complex and small-molecule inhibitors of NRF2 — e.g., brusatol (33), halofuginone (34), flumethasone (35), and digoxin (36) — that suppress NRF2 protein levels by other mechanisms may significantly enhance the chemotherapy effect and increase the survival time of patients. The reason is that the combination of these 2 small-molecule inhibitors can doubly inhibit NRF2 in gastric cancer cells compared with the use of either alone. Unfortunately, small-molecule inhibitors that specifically bind FAM117B and interfere with the FAM117B/KEAP1 complex have not yet been developed. Considering the crucial roles of FAM117B in gastric cancer, developing small-molecule inhibitors for it may provide a new approach to treating gastric cancer. In addition, future studies are needed to investigate how FAM117B protein is upregulated in gastric cancer to better understand its molecular mechanism.

## Methods

### Materials.

RPMI 1640 (11875093), F-12K (21127022), DMEM (11965092), and heat-inactivated FBS (10100147) were bought from Gibco. Anti-ubiquitin (BS62242) and anti–β-actin (AP0060) antibodies were bought from Bioworld. Anti-Ki67 (GB111141) and anti-CD31 (GB11063-2) antibodies were obtained from Servicebio. Anti-FAM117B (21768-1-AP), anti-NRF2 (66504-1-Ig), anti-KEAP1 (60027-1-Ig), anti–lamin B1 (66095-1-Ig), anti-FLAG (20543-1-AP), anti-HA (66006-2-Ig), HRP-conjugated Affinipure goat anti-mouse IgG (SA00001-1), and HRP-conjugated Affinipure goat anti-rabbit IgG (SA00001-2) antibodies were purchased from Proteintech Group. keyFluor 488 goat anti-rabbit (KGAB010) and keyFluor 488 goat anti-mouse (KGAB011) antibodies were bought from KeyGen. Cy3-conjugated goat anti-rabbit IgG (GB21303) and Cy3-conjugated goat anti-mouse IgG (GB21401) antibodies were bought from Servicebio. 5-FU (S1209), oxaliplatin (S1224), MG132 (S2619), and cycloheximide (S7418) were bought from Selleck Chemicals. MTT (M5655) was obtained from MilliporeSigma. Polybrene (C0351), puromycin (ST551), penicillin-streptomycin solution (C0222), Lipo6000 Transfection Reagent (C0526), Protein A+G Agarose (P2012), BCA Protein Assay Kit (P0012S), and ROS Assay Kit (S0033S) were bought from Beyotime. Fluorescein (FITC) Tunel Cell Apoptosis Detection Kit (G1501) and Immunohistochemistry Kits (G1215 and G1216) were obtained from Servicebio. DAPI (KGA215-50) was bought from KeyGen. RNA Isolater Total RNA Extraction Reagent (R401-01) and AceQ qPCR SYBR Green Master Mix (Q111-02) were bought from Vazyme. The pcDNA3.1-FAM117B-WT, pcDNA3.1-3 × HA-FAM117B-WT and mutant, and pcDNA3.1-3 × FLAG-KEAP1-WT and mutant plasmids were constructed by Sangon Biotech. The human gastric cancer tissue microarray (HStmA180Su20, SOB cohort) was purchased from Shanghai Outdo Biotech. Another human gastric cancer tissue microarray (AF-STC1602, AFB cohort) was purchased from AiFang Biological. A total of 30 gastric cancer tissue samples were obtained from The Affiliated Wuxi No. 2 People’s Hospital of Nanjing Medical University.

### Cell lines and cell culture.

HGC-27, AGS, and HEK293T cells were obtained from Cell Bank of the Chinese Academy of Sciences (Shanghai, China). SNU-668 cells were obtained from BLUEFBIO. HGC-27 cells were cultured in RPMI 1640 medium. AGS cells were cultured in F-12K medium. HEK293T and SNU-668 cells were cultured in DMEM. All media contained 10% heat-inactivated FBS, 100 U penicillin, and 100 μg/mL streptomycin. All cells were cultured in a humidified environment containing 5% CO_2_ at 37°C.

### Statistics.

Kaplan-Meier analysis was performed for overall survival analyses. Univariate Cox regression and multivariate Cox regression analysis were performed to analyze the relative risk of poor patient outcome. The results were expressed as mean ± SD. Statistical analysis was performed with 2-tailed unpaired Student’s *t* test (2 groups), 1-way ANOVA followed by Tukey’s test (more than 2 groups), 2-tailed Spearman test, or log-rank test. *P* less than 0.05 was considered significant difference.

### Study approval.

All animal experiments were performed in accordance with protocols approved by the Animal Ethics Committee of China Pharmaceutical University (ethics approval no. 2021-10-001). Clinical samples were collected from patients after receipt of written informed consent in accordance with a protocol approved by The Affiliated Wuxi No. 2 People’s Hospital of Nanjing Medical University.

Detailed methods are provided in Supplemental Methods online.

## Author contributions

YZ and RH designed the experiments. YZ, Yaxin Chen, and YS performed experiments. YZ, Yaxin Chen, LW, YT, and TL performed data analysis. Yigang Chen and JX collected gastric cancer tissue samples. YZ and RH wrote and edited the manuscript. YZ and RH provided funding for this study. The order of the co–first authors was assigned on the basis of their relative contributions to the study.

## Supplementary Material

Supplemental data

## Version 1 02/01/2023

Electronic publication
