# Supplementary material for: FAM117B promotes gastric cancer growth and drug resistance by targeting the KEAP1/NRF2 signaling pathway
Source: J Clin Invest. 2023 Feb 1;133(3):e158705. doi: 10.1172/JCI158705 (PMC9888386; doi:10.1172/JCI158705)
Supplement: Supplemental data [file jci-133-158705-s035.pdf]

Supplemental material

FAM117B promotes gastric cancer growth and drug resistance by targeting KEAP1-NRF2 signaling pathway

Yunjiang Zhou,[1] Yaxin Chen,[1] Yongwei Shi,[1] Leyin Wu,[1] Yingying Tan,[1] Tao Li,[1] Yigang Chen,[2] Jiazeng Xia,[2,*] and Rong Hu[1,*]

[1]State Key Laboratory of Natural Medicines, School of Basic Medicine and Clinical Pharmacy, China Pharmaceutical University, Nanjing 210009, China

[2]Department of General Surgery, The Affiliated Wuxi No.2 People's Hospital of Nanjing Medical University, Wuxi 214002, China

Authorship note: YZ and YC contributed equally to this work.

***Corresponding Author:**

Rong Hu, State Key Laboratory of Natural Medicines, School of Basic Medicine and Clinical Pharmacy, China Pharmaceutical University, Nanjing 210009, China. Phone: 86.025.83271089; Email: ronghu@cpu.edu.cn.

Jiazeng Xia, Department of General Surgery, The Affiliated Wuxi No.2 People's Hospital of Nanjing Medical University, Wuxi 214002, China. Phone: 86.0510.68562007; Email: jiazengxiawuxi@njmu.edu.cn.

Supplemental methods

Western blot assay. Cells and tissues were lysed in RIPA lysis buffer with 1mM PMSF for 1 h on ice and centrifuged at 13,000 rpm for 30 min at 4 °C. The concentrations of total protein were measured by using BCA protein assay kit. Equal amounts of protein were separated by SDS-PAGE and transferred onto polyvinylidene difluoride (PVDF) membranes. Membranes were blocked with 3% BSA in PBS at 37 °C for 2 h and incubated with indicated antibodies overnight at 4 °C. Membranes were then washed three times with PBST followed by incubation with HRP-conjugated secondary antibodies for 1 h at 37 °C. The signals were analyzed using the ECL chemiluminescence detection system (Tanon, Shanghai, China) and Image J software was used to quantify the intensity of the bands.

MTT assay. To determine the cytotoxicity of chemotherapeutic drugs, cells transfected with the indicated shRNA or overexpression plasmids were plated into 96-well plates at approximately 5,000 cells/well in 100 μL medium, then treated with various concentrations of chemotherapeutic drugs for 48 h. Cell viability was assessed with the MTT assay following the manufacturer's protocol. Cell survival ratio was calculated using Atreated/Acontrol × 100%, where Atreated and Acontrol were the absorbance from treated and control cells after 48 h incubation, respectively. The IC_{50} was taken as the concentration that caused 50% inhibition of cell proliferation and was calculated by SPSS statistical software.

Cell growth assay. Cells transfected with the indicated shRNA or overexpression plasmids were seeded into 6-well plates. The number of viable cells per well were counted daily.

Colony formation assay. Cells transfected with the indicated shRNA or overexpression plasmids were seeded into 6-well plates and incubated for 14 days. Cells were then fixated with 4% formaldehyde and stained with 0.5% crystal violet. Only colonies with more than 50 cells were counted.

Plasmids transfection and shRNA interference. For plasmids transfection, cells were seeded in a 6-well plate and maintained in medium without antibiotics. After 24 h, the plasmids and Lipo6000 Transfection Reagent were diluted and mixed in serum-free medium according to the manufacturer's instruction. Then, the plasmids-transfection reagent complexes were added into wells. The medium was replaced after 4 h incubation and the cells were collected to perform western blot analysis after 36 h culture. For shRNA interference, FAM117B, NRF2 and KEAP1 shRNAs lentiviral particles were diluted in OptiMEM containing 6 μg/ml polybrene, and then were added to cells. After 3 days,

5 μg/ml of puromycin were used to select transfected cells. Cells transfected with the shRNAs lentiviral particles were seeded into six-well plates and western blot analysis were used to detect the protein levels of FAM117B, NRF2, and KEAP1.

RNA extraction and qPCR assays. Total RNA was isolated using the RNA isolater Total RNA Extraction Reagent according to the manufacturer's protocol. The concentration and purity of the extracted RNA were measured with the optical densities at 260 and 280 nm. RNA samples were reverse transcribed to cDNA and subjected to quantitative PCR, which was performed with the Light-Cycler_96 RealTime PCR System (Roche) using AceQ qPCR SYBR Green Master Mix. The primer sequences used in this study were shown in Supplemental Table 6.

CHX-chase assay. Degradation half-life of NRF2 was analyzed by CHX-chase assay. Briefly, cells transfected with the indicated shRNA or overexpression plasmids were incubated with 25 μM of cycloheximide (CHX) to inhibit protein synthesis. Total cell lysates were collected at 0, 15, 30, 45, and 60 min after following treatment with CHX and subjected to western blot analysis with indicated antibodies. The intensity of the bands were quantified by using Image J software.

Co-immunoprecipitation assay. Immunoprecipitation assay was performed using a standard protocol. Briefly, the cell lysates were incubated with indicated antibodies overnight at 4°C, and then incubated with protein A+G agarose beads for another 4 h at 4°C. Immunoprecipitated proteins were analyzed by western blot with indicated antibodies.

GST pull-down assay. For the GST pull-down assay, the glutathione sepharose beads conjugated with 500 ng GST or GST-KEAP1-WT protein were incubated with 200 ng purified FAM117B in GST-binding buffer at 4°C for 24 h, followed by washing 3 times for 10 minutes each with GST-washing buffer at 4°C. The samples were analyzed by western blot with indicated antibodies.

Measurement of intracellular ROS level. Intracellular ROS level was detected by using ROS assay kit according to the manufacturer's instructions. The fluorescence intensity was measured by using microplate reader at Ex./Em. = 488/525 nm.

MST assay. The different concentrations of FAM117B, NRF2, and NRF2$^{\Delta DLG}$ proteins were incubated with GFP-tagged KEAP1 protein for 5 min at room temperature in assay buffer containing 0.05% Tween 20. The fluorescent signal were detected by Monolith NT.115 (NanoTemper, Munich, Germany). The Kd value was calculated by fitting a standard binding curve.

Animal experiments. Female BALB/c nude mice (6 weeks old) were purchased from Beijing Vital River Laboratory Animal Technology Co., Ltd (Beijing, China). All protocols for mice were

approved by the Animal Ethics Committee of China Pharmaceutical University (Ethic approval number: 2021-10-001). Cells stably transfected with the indicated shRNA or overexpression plasmids were injected into subdermal space of mice (1×10^7 cells/per mouse). Tumor volume of mice were measured every four days. Tumor volume = $(a \times a \times b)/2$ (a, the smallest diameter; b, the largest diameter). For 5-FU treatment, mice were injected with cells stably transfected with the indicated shRNA or overexpression plasmids in the subdermal space. Once the tumors reached 80-100 mm^3, the mice were treated with PBS or 5-FU (40 mg/kg, 5 times, intraperitoneally) for 20 days. Tumor volume of mice were measured every four days.

Immunohistochemistry assay. Immunohistochemistry (IHC) staining was performed by using immunohistochemistry kit according to the manufacturer's protocol. Briefly, 5 μm-thick paraffin sections were deparaffinized, rehydrated and washed 3 times with PBS. Antigen repair solution was used to repair the antigen of tissues. Then, tissue sections were treated with 3% hydrogen peroxide at 37°C for 25 min, blocked with 3% BSA at 37°C for 1 h, and incubated with primary antibodies at 4°C overnight. HRP-conjugated secondary anti-rabbit or HRP-conjugated secondary anti-mouse antibody was added and incubated at room temperature for 1 h. The tissue sections were stained with DAB substrate and counterstained with hematoxylin. All images were acquired by an inverted microscope (Nikon, Japan).

Immunofluorescence and TUNEL assays. For cellular immunofluorescence assay, cells were fixed in 4% paraformaldehyde for 20 min, and washed with PBS for 3 times. Then, cells were incubated with Triton X-100 for 5 min, blocked with 3% BSA at 37°C for 1 h, and incubated with primary antibodies at 4°C overnight. The cells were washed with PBS for 3 times and incubated with keyFluor 488 Goat Anti-Rabbit, keyFluor 488 Goat Anti-Mouse, Cy3 conjugated Goat Anti-Rabbit IgG, or Cy3 conjugated Goat Anti-Mouse IgG antibody for 1 h. Next, the cells were washed with PBS for 3 times and incubated with DAPI. For tissue immunofluorescence assay, 5 μm-thick paraffin sections were deparaffinized, rehydrated and washed with PBS for 3 times. Antigen repair solution was used to repair the antigen of tissues. Then, the tissue sections were incubated with Triton X-100 for 5 min, treated with 3% hydrogen peroxide at 37°C for 25 min, blocked with 3% BSA at 37°C for 1 h, and incubated with primary antibodies at 4°C overnight. The tissue sections were washed with PBS for 3 times and incubated with Cy3 conjugated Goat Anti-Rabbit IgG antibody for 1 h. Next, the tissue sections were washed with PBS for 3 times and incubated with DAPI. For TUNEL assay, TUNEL apoptosis detection kit was used to confirm in situ cell apoptosis on 5 μm-thick paraffin

sections according to the manufacturer's instructions. All images were acquired by an inverted microscope (Nikon, Japan).

Human gastric cancer tissue microarrays. The human gastric cancer tissue microarray (contained 104 gastric tumor tissues and 76 adjacent normal tissues) was purchased from Shanghai Outdo Biotech (SOB cohort). In SOB cohort, all of the patients had undergone surgical treatment, but the information about their chemotherapy were missing. Another human gastric cancer tissue microarray (contained 80 gastric tumor tissues and 80 adjacent normal tissues) obtained from AiFang Biological was used as an independent cohort (AFB cohort) to validate the relationship between FAM117B, NRF2 and clinical outcome. In AFB cohort, all of the patients had undergone surgical treatment and the majority of these patients (76/80, 4 patients had no information on chemotherapy) received adjuvant chemotherapy alone (platinum, fluorouracil, anthracycline, and paclitaxel) or combined chemotherapy. All patients had been pathologically diagnosed with gastric cancer. IHC staining was used to analyze the protein levels of FAM117B and NRF2 in gastric tumor tissues and matched adjacent normal tissues. Each specimen was assigned a score according to the intensity of the staining (no staining = 0, weak staining = 1, moderate staining = 2, strong staining = 3) and the extent of stained cells (0% = 0, 1-24% = 1, 25-49% = 2, 50-74% = 3, 75-100% = 4). When the stain was homogenous, the IHC score was determined by multiplying the intensity score with the extent score of stained cells. When the stain was heterogeneous, we scored it as follows: each component was scored independently and summed for the results. A score of 6 or less was defined as a low IHC score, and a score higher than 6 was defined as a high IHC score. All of staining was assessed by pathologist blinded to the origination of the samples and subject outcome.

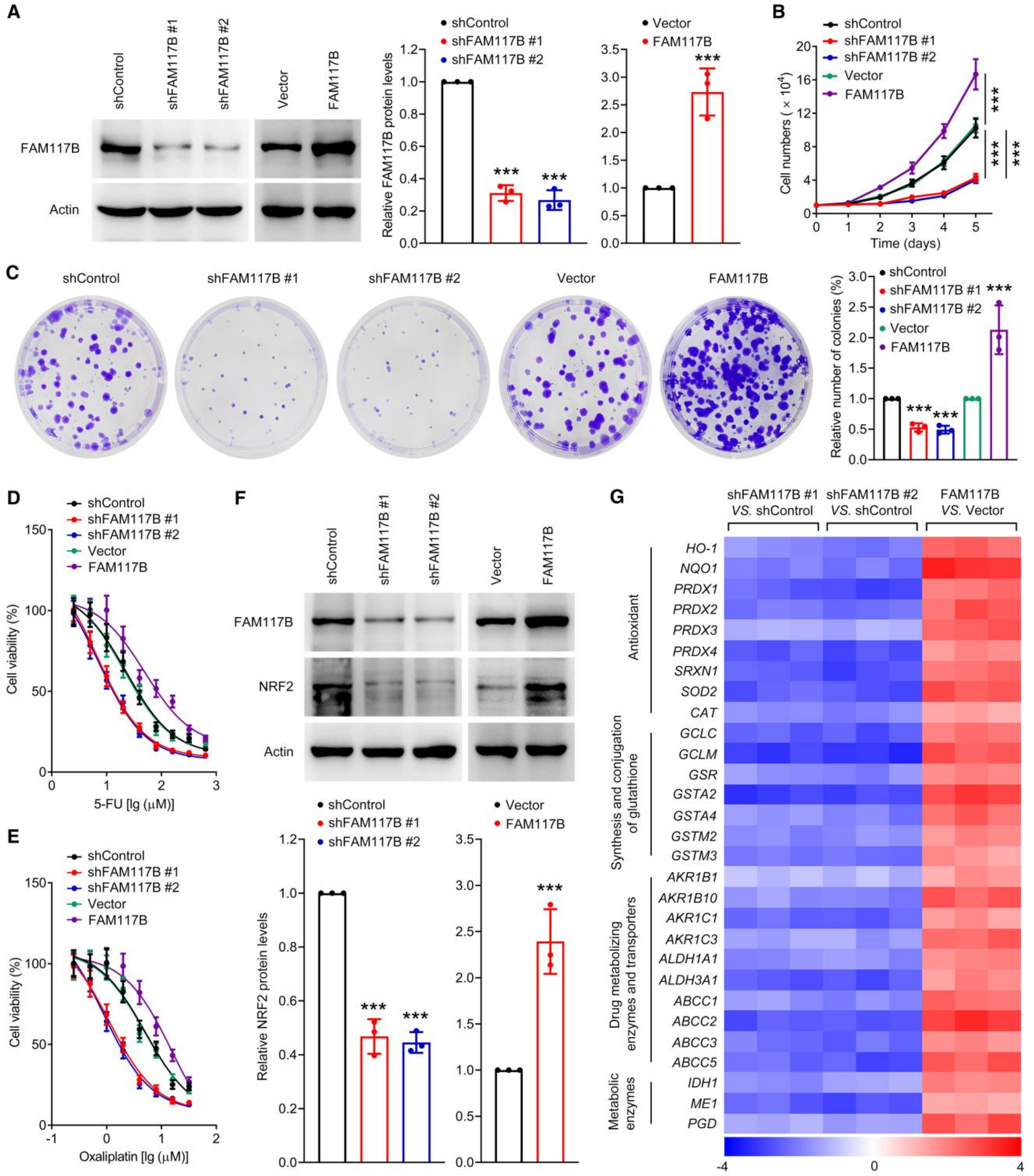

Supplemental Figure 1. FAM117B promotes the growth and chemoresistance of SNU-668 cells, and activates KEAP1-NRF2 signaling in SNU-668 cells. (**A**) The FAM117B protein levels in SNU-668 cells transfected with shFAM117B (#1 and #2) or overexpression plasmids (n = 3 independent experiments). (**B**) Cell growth ability of SNU-668 cells with FAM117B knockdown or overexpression (n = 3 independent experiments). (**C**) Colony formation ability of SNU-668 cells with FAM117B knockdown or overexpression (n = 3 independent experiments). (**D and E**) Therapeutic efficacy of 5-FU and oxaliplatin on SNU-668 cells with FAM117B knockdown or overexpression (n

= 3 independent experiments). (**F**) The protein levels of NRF2 in SNU-668 cells with FAM117B knockdown or overexpression (*n* = 3 independent experiments). (**G**) The mRNA levels of NRF2 target genes in SNU-668 cells with FAM117B knockdown or overexpression (*n* = 3 independent experiments). The colors of the heatmap represent values of $-\Delta\Delta Ct$. Data are shown as mean ± SD. ***$P < 0.001$ means significant difference *vs.* shControl or Vector group. The statistical significance was calculated using 2-tailed unpaired Student's *t* test (2 groups) or one-way ANOVA, followed by Tukey's test (more than 2 groups).

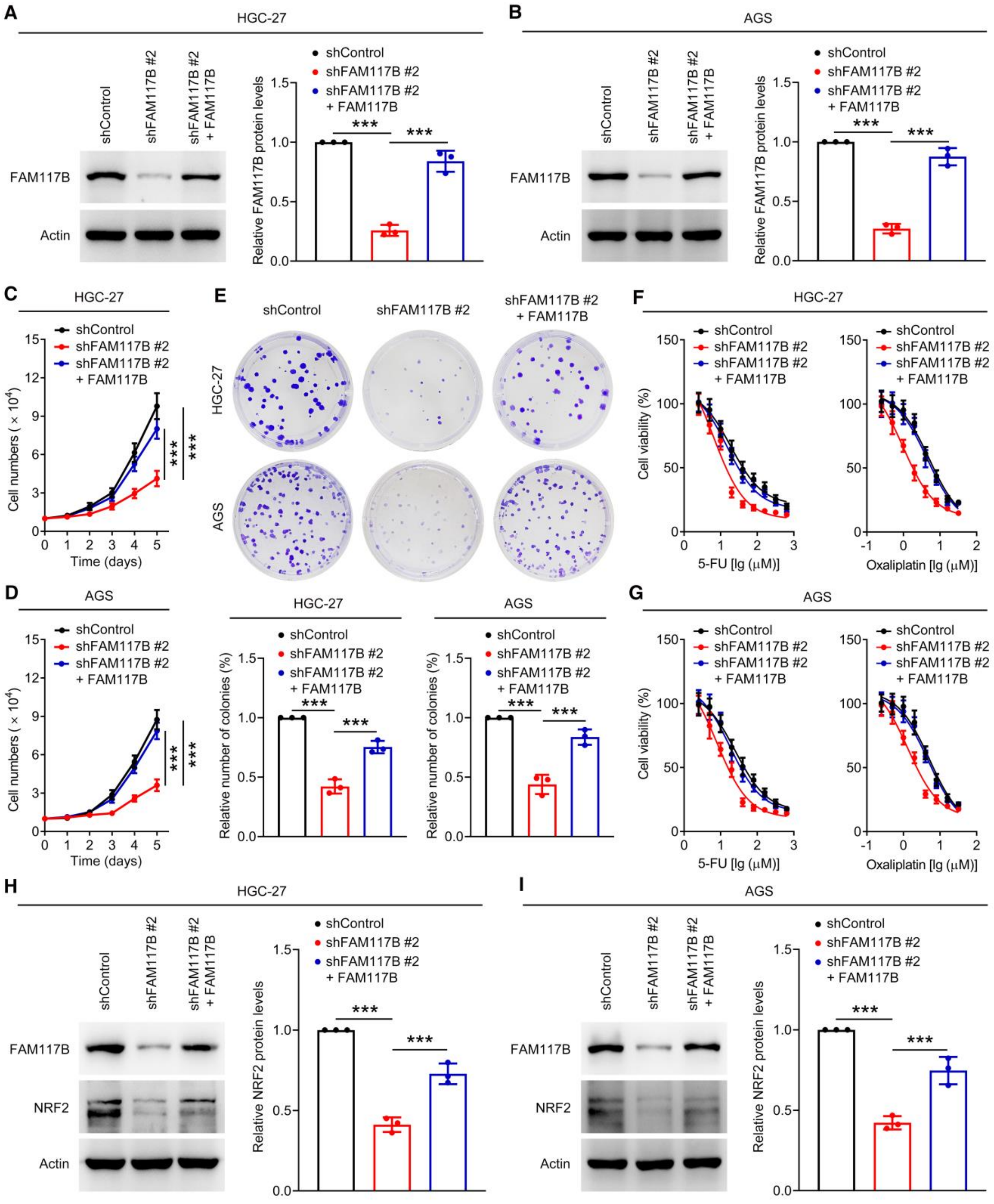

Supplemental Figure 2. Restoring FAM117B expression can abolish FAM117B knockdown-induced gastric cancer cell growth inhibition, chemosensitization, and NRF2 protein suppression. (**A** and **B**) The FAM117B protein levels in HGC-27 and AGS cells transfected with shControl, shFAM117B #2, and shFAM117B #2 + FAM117B (*n* = 3 independent experiments). (**C** and **D**) Cell growth ability of HGC-27 and AGS cells transfected with shControl, shFAM117B #2, and shFAM117B #2 + FAM117B (*n* = 3 independent experiments). (**E**) Colony formation ability of

HGC-27 and AGS cells transfected with shControl, shFAM117B #2, and shFAM117B #2 + FAM117B (*n* = 3 independent experiments). (**F** and **G**) Therapeutic efficacy of 5-FU and oxaliplatin on HGC-27 and AGS cells transfected with shControl, shFAM117B #2, and shFAM117B #2 + FAM117B (*n* = 3 independent experiments). (**H** and **I**) The protein levels of NRF2 in HGC-27 and AGS cells transfected with shControl, shFAM117B #2, and shFAM117B #2 + FAM117B (*n* = 3 independent experiments). Data are shown as mean ± SD. ***$P < 0.001$ means significant difference *vs.* shFAM117B #2 group. The statistical significance was calculated using one-way ANOVA, followed by Tukey's test.

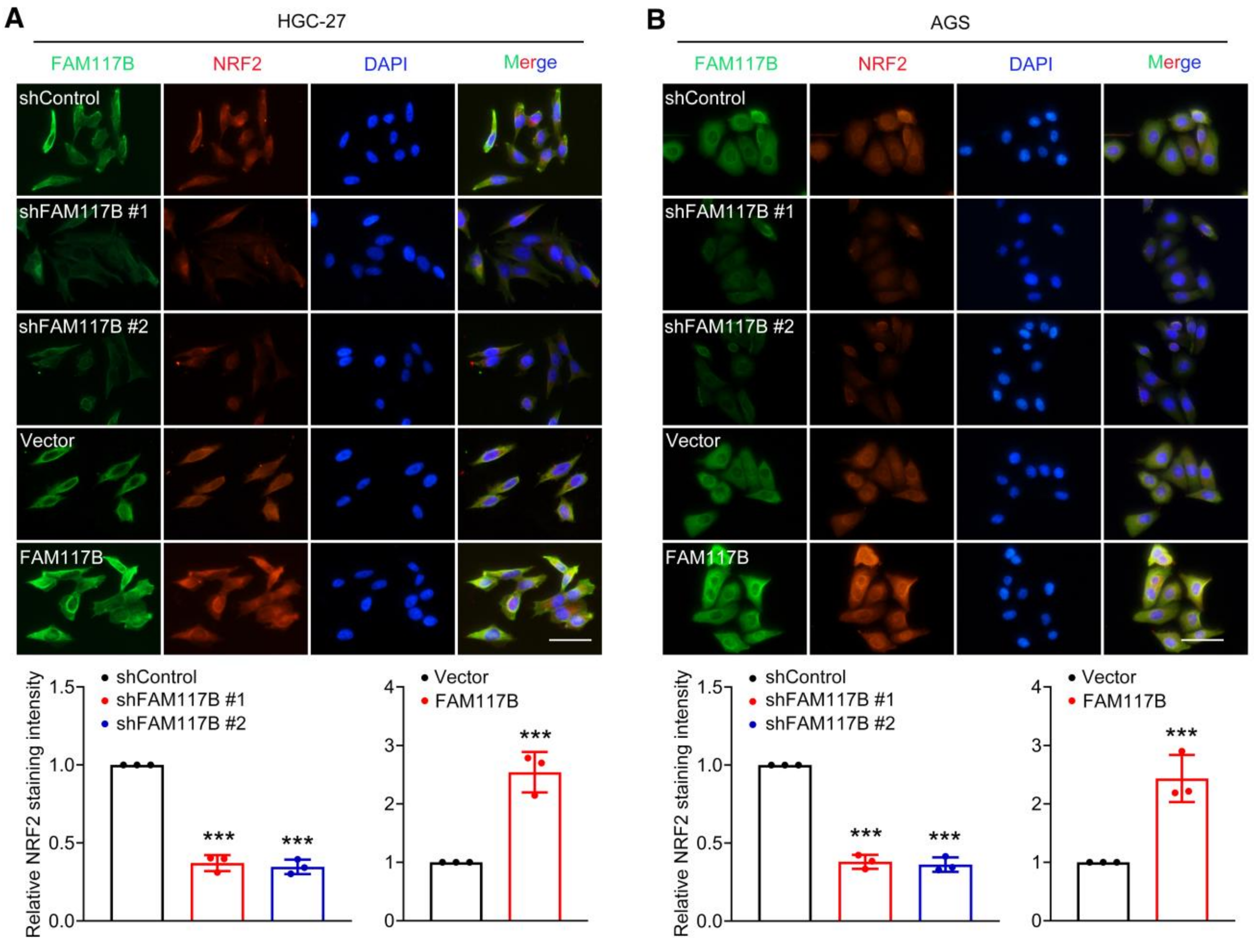

Supplemental Figure 3. The protein levels of NRF2 in HGC-27 and AGS cells with FAM117B knockdown or overexpression. (**A**) Immunofluorescence assay in HGC-27 cells with FAM117B knockdown or overexpression (*n* = 3 independent experiments). Scale bars = 50 μm. (**B**) Immunofluorescence assay in AGS cells with FAM117B knockdown or overexpression (*n* = 3 independent experiments). Scale bars = 50 μm. Data are shown as mean ± SD. ***$P < 0.001$ means significant difference *vs.* shControl or Vector group. The statistical significance was calculated using 2-tailed unpaired Student's *t* test (2 groups) or one-way ANOVA, followed by Tukey's test (more than 2 groups).

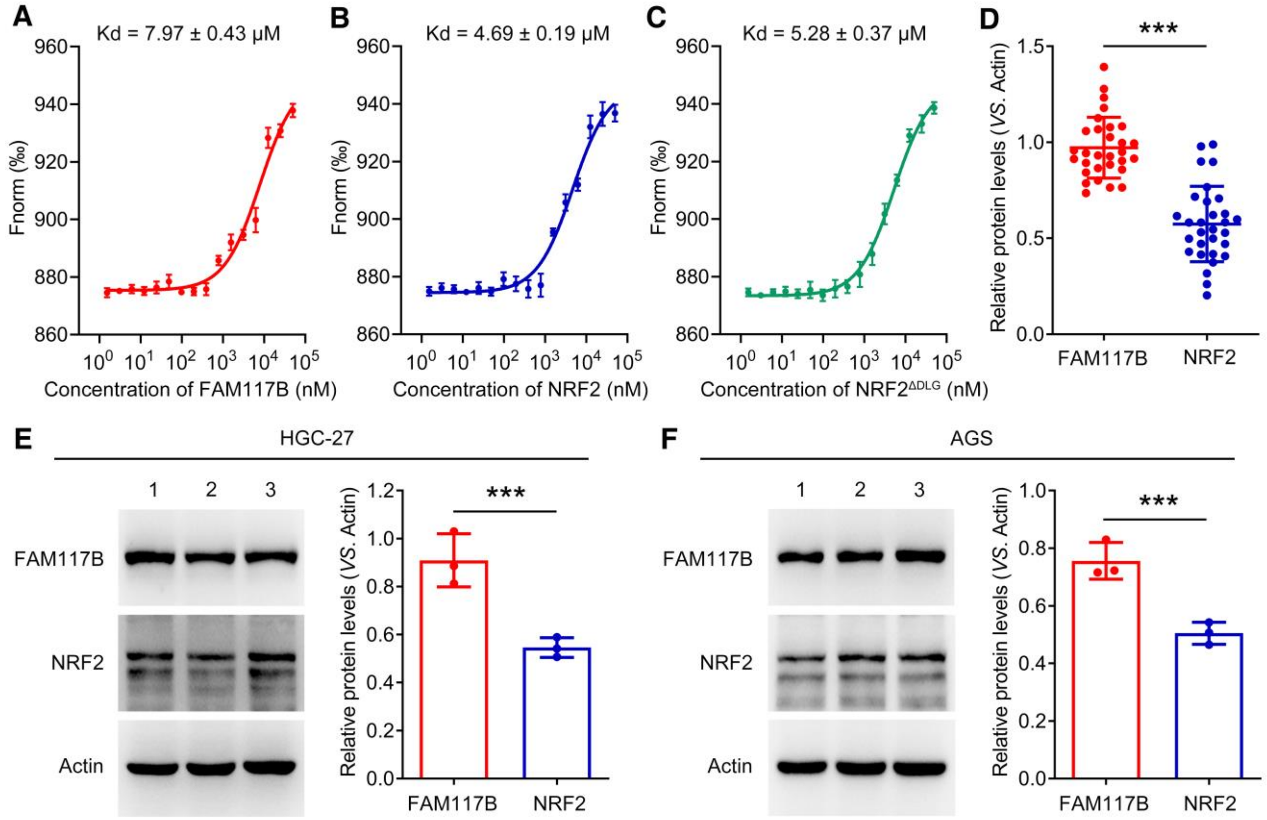

Supplemental Figure 4. (**A**) Binding affinity between FAM117B and KEAP1 proteins (*n* = 3 independent experiments). (**B**) Binding affinity between NRF2 and KEAP1 proteins (*n* = 3 independent experiments). (**C**) Binding affinity between NRF2$^{\Delta DLG}$ (DLG motif deletion of NRF2) and KEAP1 proteins (*n* = 3 independent experiments). (**D**) The protein levels of FAM117B and NRF2 in tumor tissues of gastric cancer patients (*n* = 30). (**E**) The protein levels of FAM117B and NRF2 in HGC-27 cells (*n* = 3 independent experiments). (**F**) The protein levels of FAM117B and NRF2 in AGS cells (*n* = 3 independent experiments). Data are shown as mean ± SD. ***$P < 0.001$ means significant difference. The statistical significance was calculated using 2-tailed unpaired Student's *t* test.

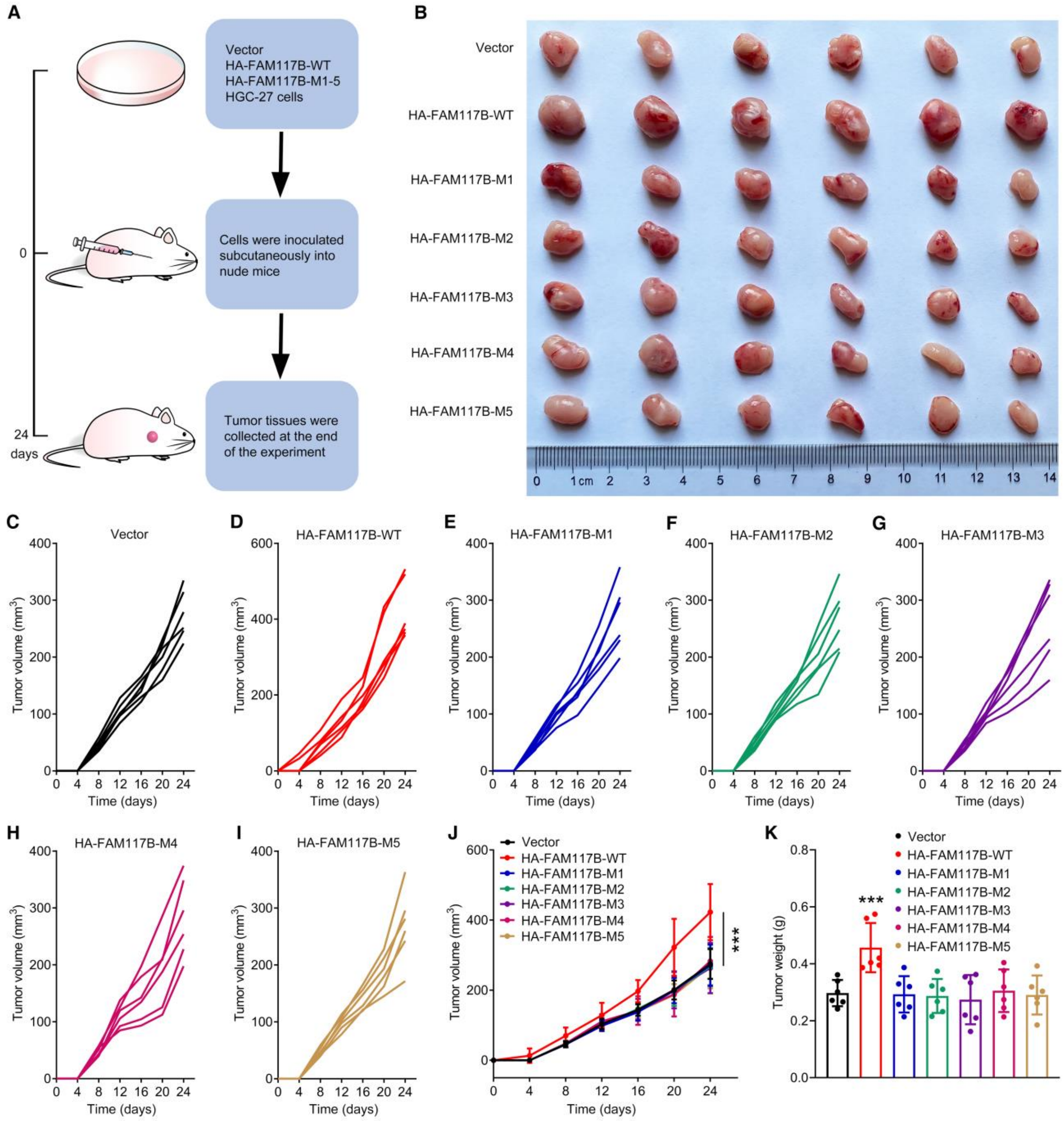

Supplemental Figure 5. The ETGE motif of FAM117B promotes the growth of HGC-27 cell-derived xenografts. (**A**) Schematic diagram of the in vivo study. (**B**) Image of tumors (n = 6 per group). (**C-J**) Growth curves of tumor volume (n = 6 per group). (**K**) Tumor weight (n = 6 per group). Data are shown as mean ± SD. ***P < 0.001 means significant difference *vs.* Vector group. The statistical significance was calculated using one-way ANOVA, followed by Tukey's test.

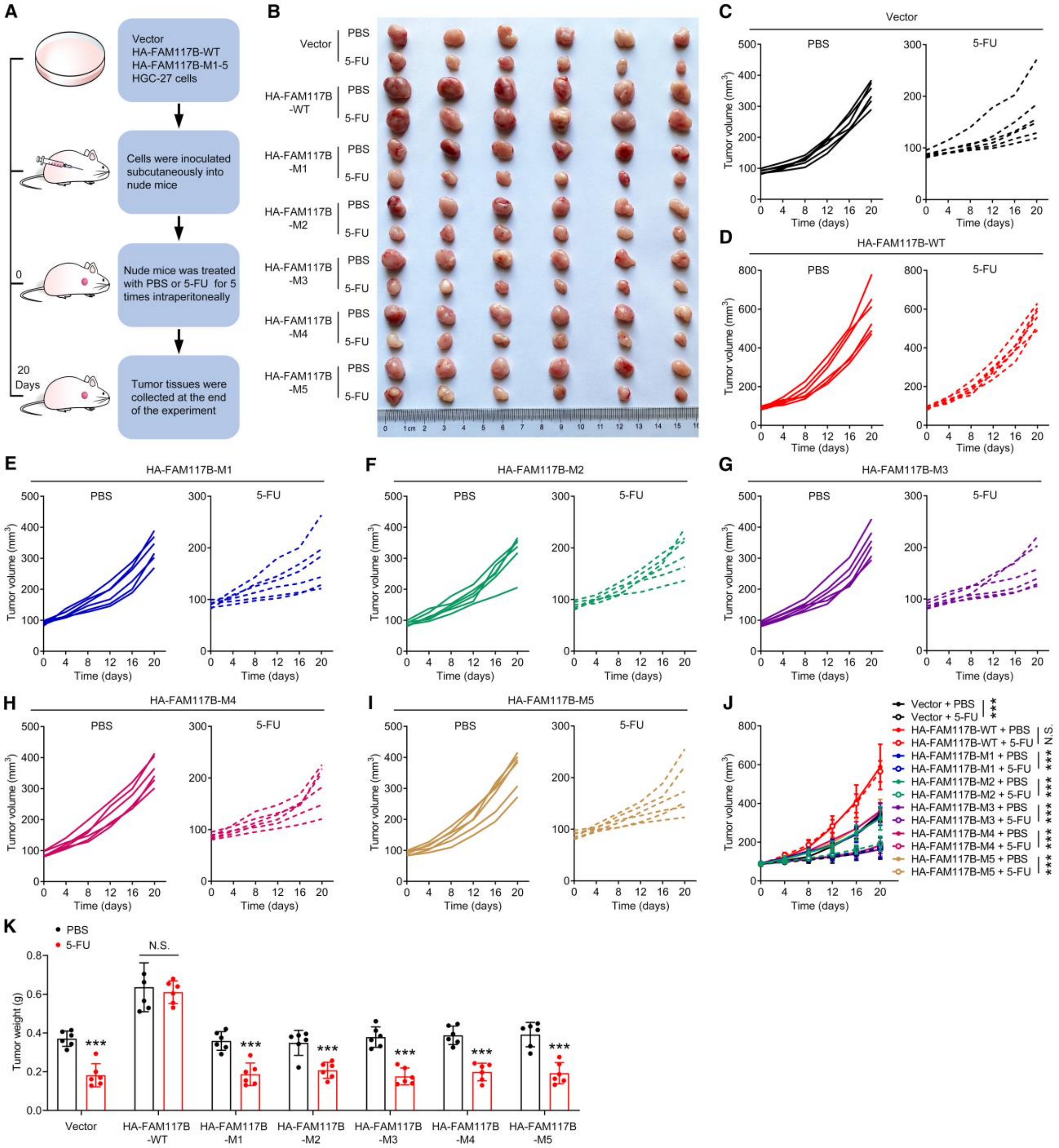

Supplemental Figure 6. The ETGE motif of FAM117B promotes the chemoresistance of HGC-27 cell-derived xenografts. (**A**) Schematic diagram of the in vivo study. (**B**) Image of tumors (n = 6 per group). (**C-J**) Growth curves of tumor volume (n = 6 per group). (**K**) Tumor weight (n = 6 per group). Data are shown as mean ± SD. ***P < 0.001 means significant difference *vs.* PBS group. N.S. means no significant difference. The statistical significance was calculated using one-way ANOVA, followed by Tukey's test.

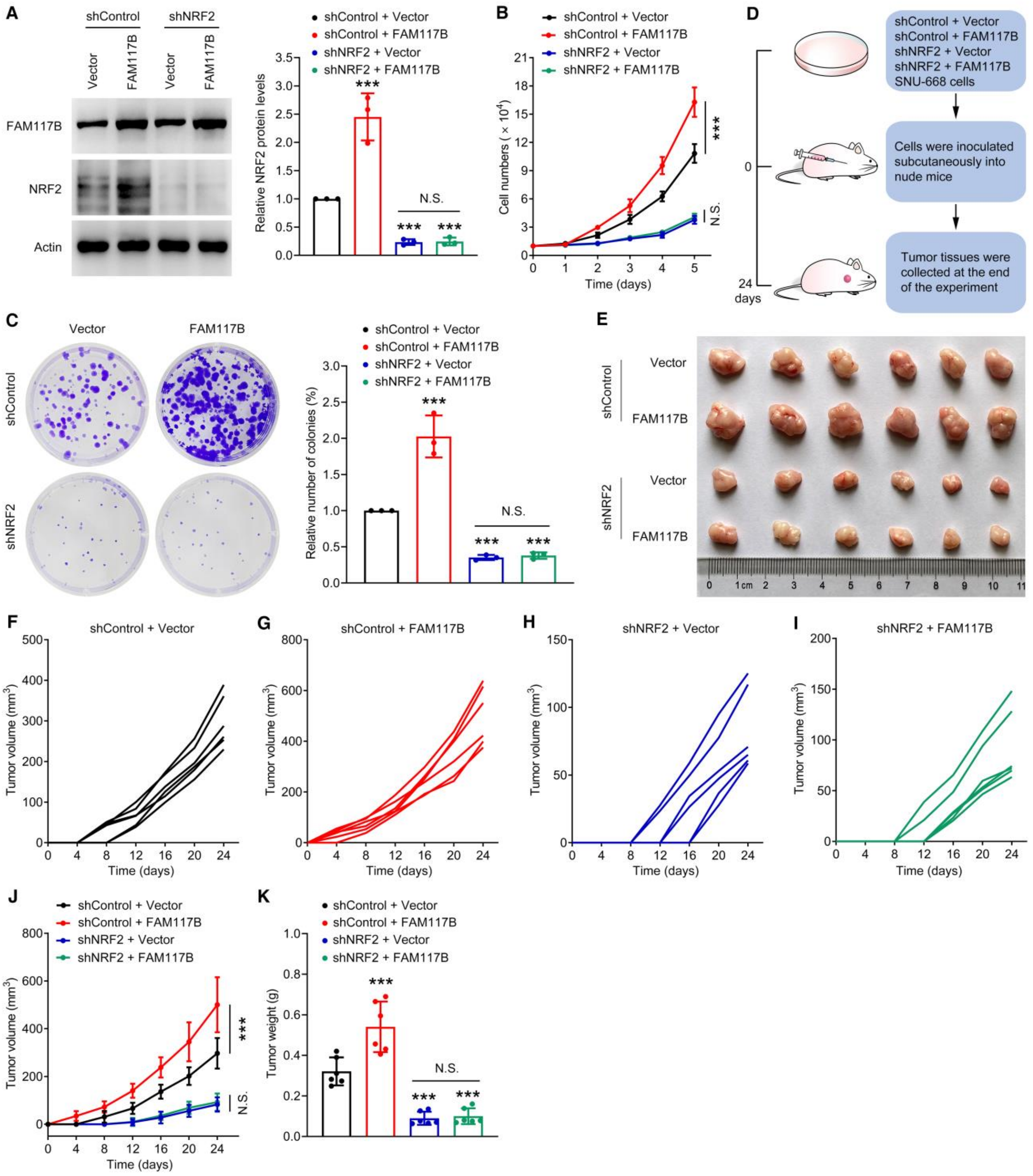

Supplemental Figure 7. FAM117B promotes the growth of SNU-668 cells via regulating KEAP1-NRF2 signaling. (**A**) The protein levels of NRF2 in SNU-668 cells transfected with shControl + Vector, shControl + FAM117B, shNRF2 + Vector, and shNRF2 + FAM117B (*n* = 3 independent experiments). (**B**) Cell growth ability of SNU-668 cells transfected with shControl + Vector, shControl + FAM117B, shNRF2 + Vector, and shNRF2 + FAM117B (*n* = 3 independent experiments). (**C**) Colony formation ability of SNU-668 cells transfected with shControl + Vector, shControl + FAM117B, shNRF2 + Vector, and shNRF2 + FAM117B (*n* = 3 independent experiments). (**D**) Schematic diagram of the in vivo study. (**E**) Image of tumors (*n* = 6 per group). (**F-J**) Growth curves

of tumor volume (*n* = 6 per group). (**K**) Tumor weight (*n* = 6 per group). Data are shown as mean ± SD. $^{***}P < 0.001$ means significant difference *vs.* shControl + Vector group. N.S. means no significant difference. The statistical significance was calculated using one-way ANOVA, followed by Tukey's test.

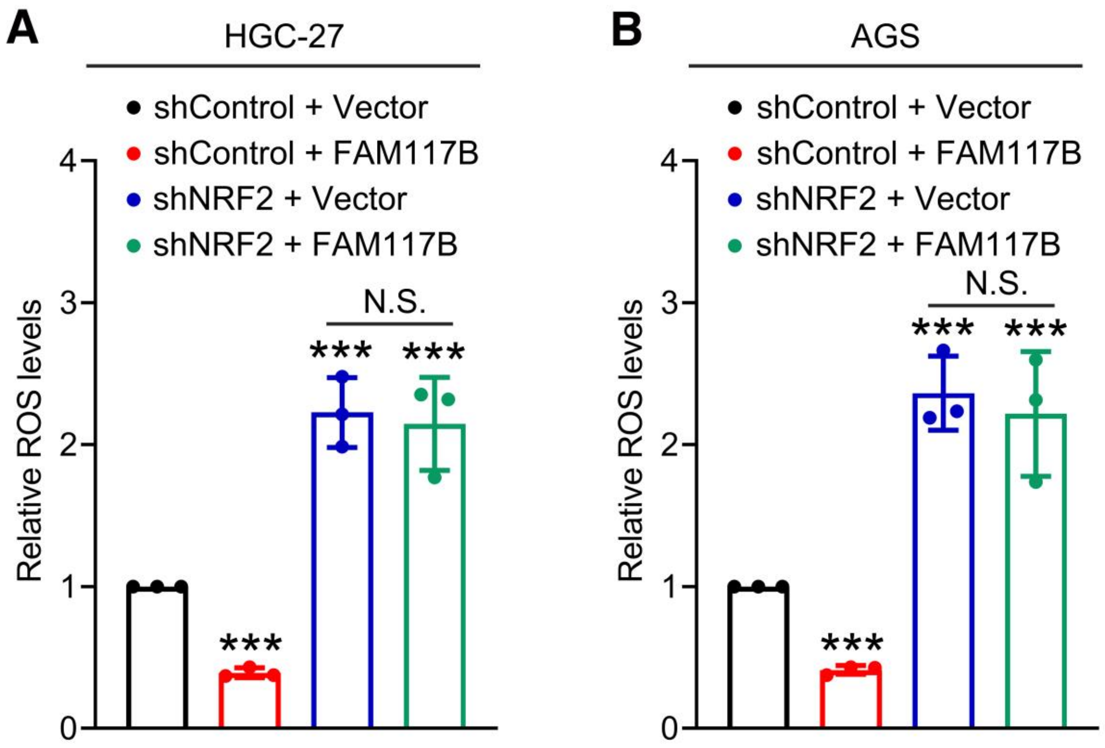

Supplemental Figure 8. FAM117B induces NRF2 to regulate ROS levels in gastric cancer cells. (**A**) The ROS levels in HGC-27 cells transfected with shControl + Vector, shControl + FAM117B, shNRF2 + Vector, and shNRF2 + FAM117B (*n* = 3 independent experiments). (**B**) The ROS levels in AGS cells transfected with shControl + Vector, shControl + FAM117B, shNRF2 + Vector, and shNRF2 + FAM117B (*n* = 3 independent experiments). Data are shown as mean ± SD. ***$P < 0.001$ means significant difference *vs.* shControl + Vector group. N.S. means no significant difference. The statistical significance was calculated using one-way ANOVA, followed by Tukey's test.

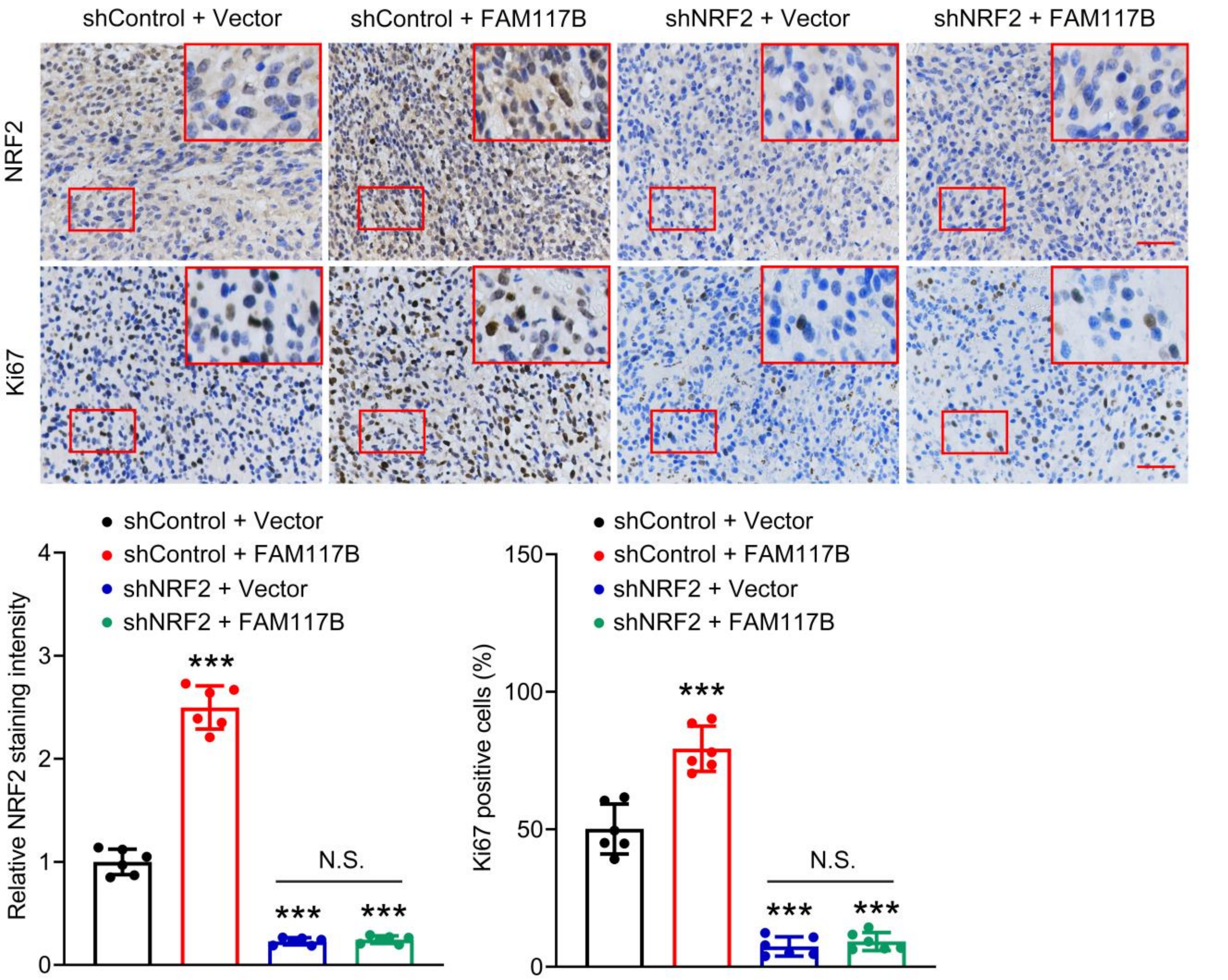

Supplemental Figure 9. The NRF2 protein levels and positive rates of Ki67 in shControl + Vector, shControl + FAM117B, shNRF2 + Vector, and shNRF2 + FAM117B-HGC-27 cell-derived xenografts. Scale bar = 50 μm. Data are shown as mean ± SD, $n = 6$ per group. ***$P < 0.001$ means significant difference *vs.* shControl + Vector group. N.S. means no significant difference. The statistical significance was calculated using one-way ANOVA, followed by Tukey's test.

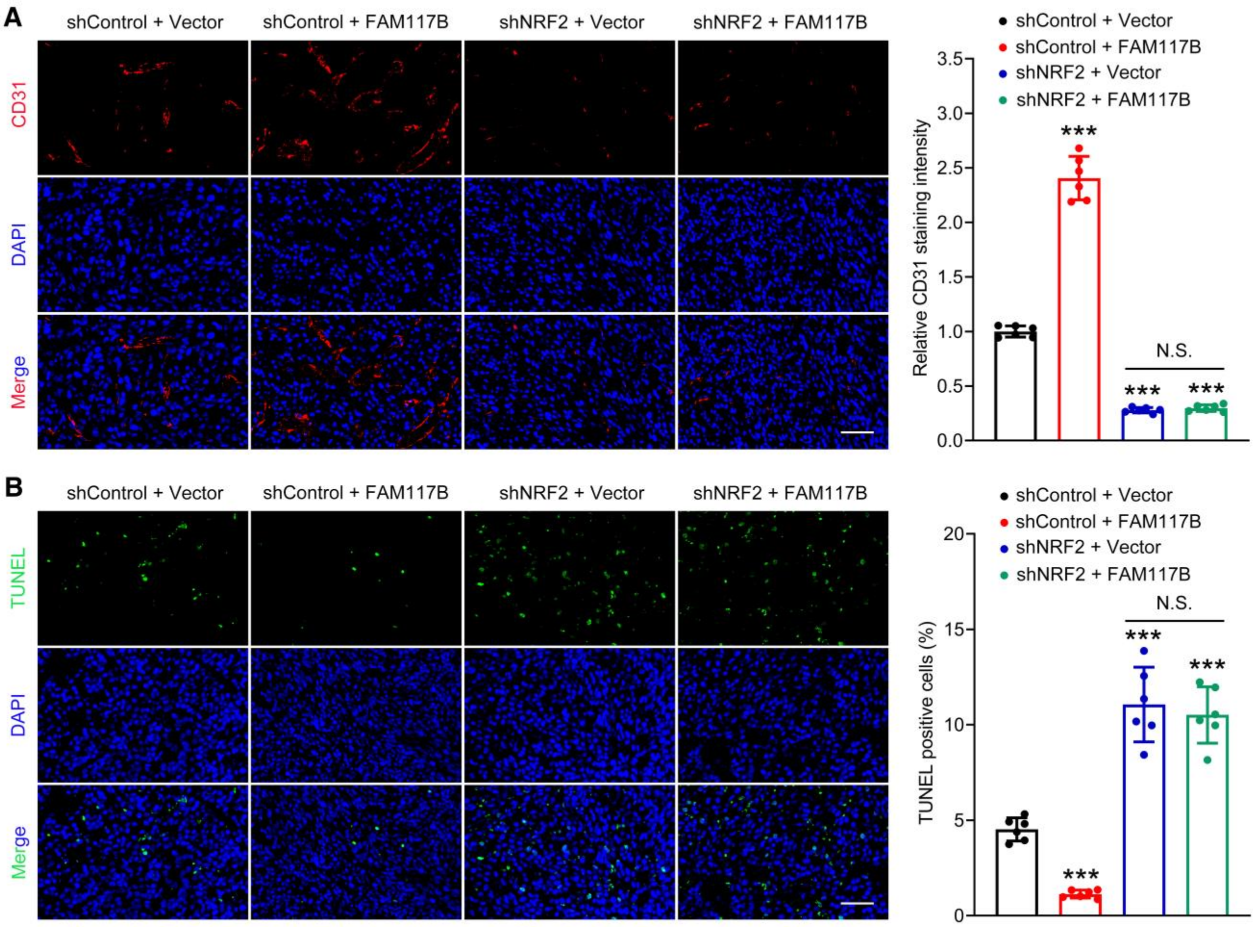

Supplemental Figure 10. FAM117B promotes angiogenesis and inhibits cell apoptosis in HGC-27 cell-derived xenografts by regulating KEAP1-NRF2 signaling. (**A**) The protein levels of CD31 in shControl + Vector, shControl + FAM117B, shNRF2 + Vector, and shNRF2 + FAM117B-HGC-27 cells-derived xenografts (*n* = 6 per group). Scale bars = 50 μm. (**B**) The number of TUNEL positive cells in shControl + Vector, shControl + FAM117B, shNRF2 + Vector, and shNRF2 + FAM117B-HGC-27 cells-derived xenografts (*n* = 6 per group). Scale bars = 50 μm. Data are shown as mean ± SD. ****P* < 0.001 means significant difference *vs.* shControl + Vector group. N.S. means no significant difference. The statistical significance was calculated using one-way ANOVA, followed by Tukey's test.

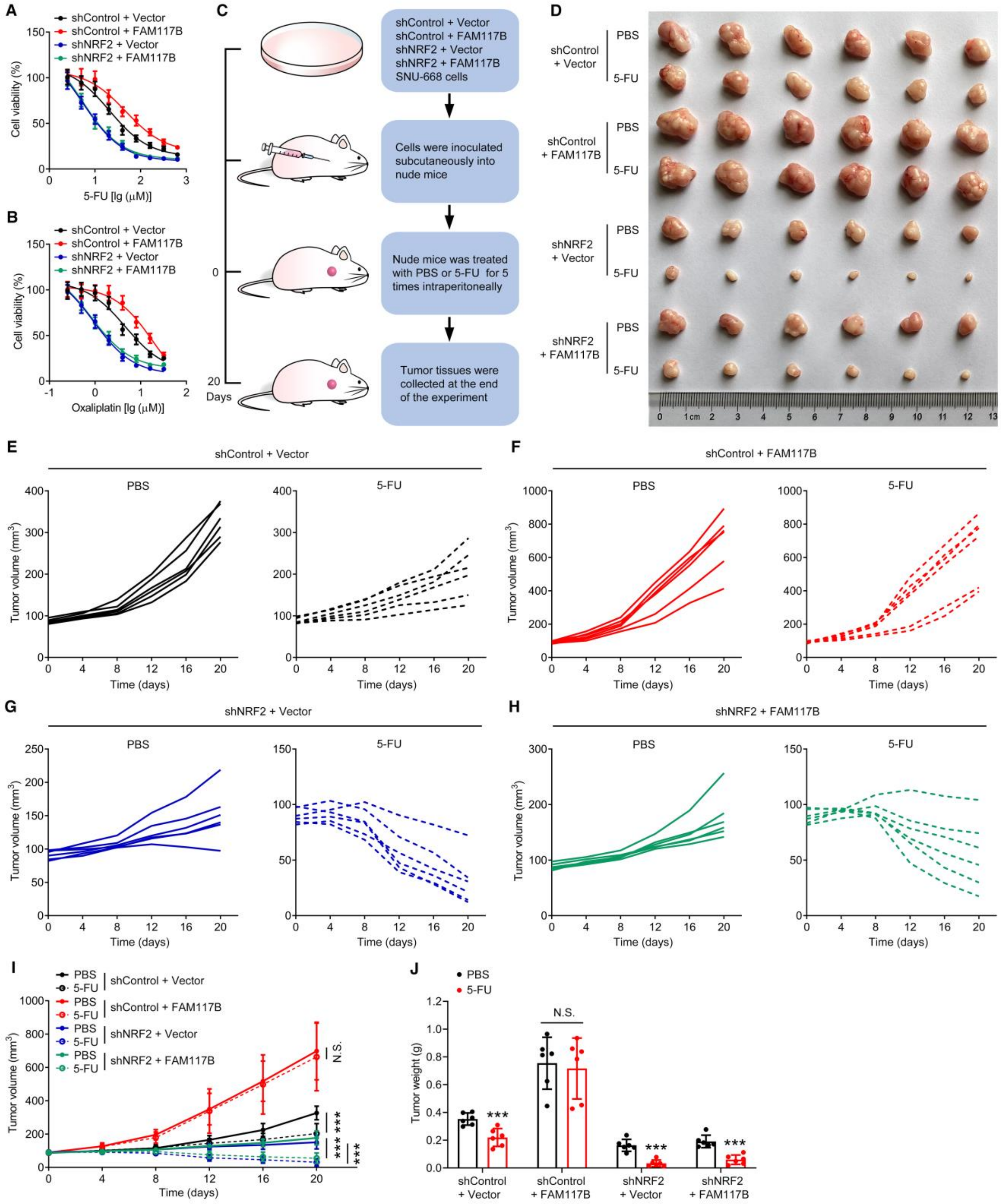

Supplemental Figure 11. FAM117B promotes the chemoresistance of SNU-668 cells via regulating KEAP1-NRF2 signaling. (**A** and **B**) Therapeutic efficacy of 5-FU and oxaliplatin on SNU-668 cells transfected with shControl + Vector, shControl + FAM117B, shNRF2 + Vector, and shNRF2 + FAM117B (*n* = 3 independent experiments). (**C**) Schematic diagram of the in vivo study. (**D**) Image of tumors (*n* = 6 per group). (**E-I**) Growth curves of tumor volume (*n* = 6 per group). (**J**) Tumor weight (*n* = 6 per group). Data are shown as mean ± SD. ***P < 0.001 means significant

difference *vs.* PBS group. N.S. means no significant difference. The statistical significance was calculated using one-way ANOVA, followed by Tukey’s test.

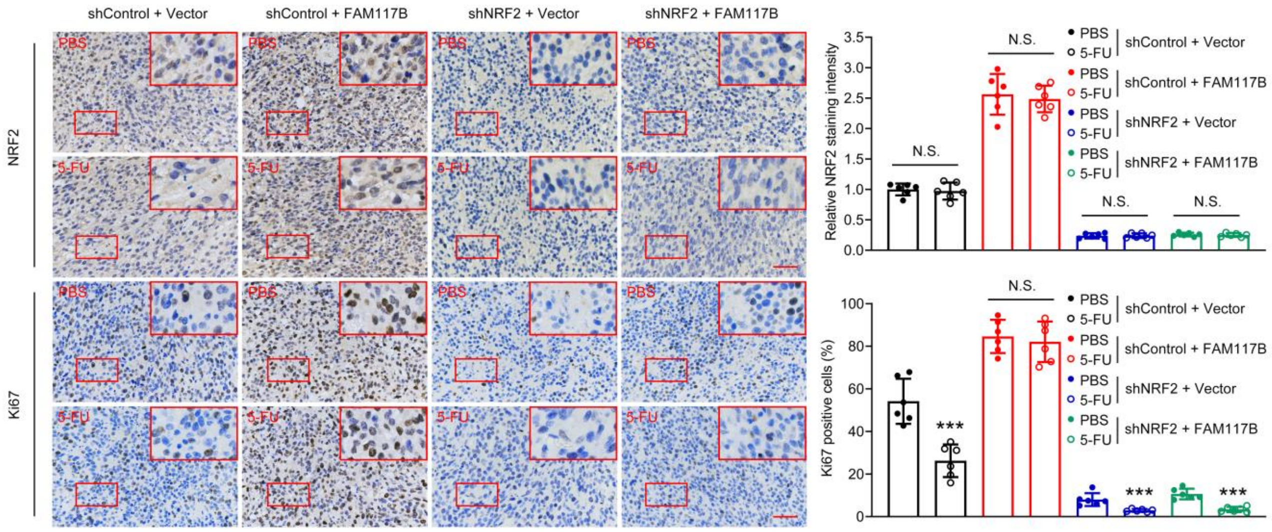

Supplemental Figure 12. The NRF2 protein levels and positive rates of Ki67 in HGC-27 cell-derived xenograft tissues of shControl + Vector + PBS, shControl + Vector + 5-FU, shControl + FAM117B + PBS, shControl + FAM117B + 5-FU, shNRF2 + Vector + PBS, shNRF2 + Vector + 5-FU, shNRF2 + FAM117B + PBS, and shNRF2 + FAM117B + 5-FU groups. Scale bar = 50 μm. Data are shown as mean ± SD, $n = 6$ per group. ***$P < 0.001$ means significant difference *vs.* PBS group. N.S. means no significant difference. The statistical significance was calculated using one-way ANOVA, followed by Tukey's test.

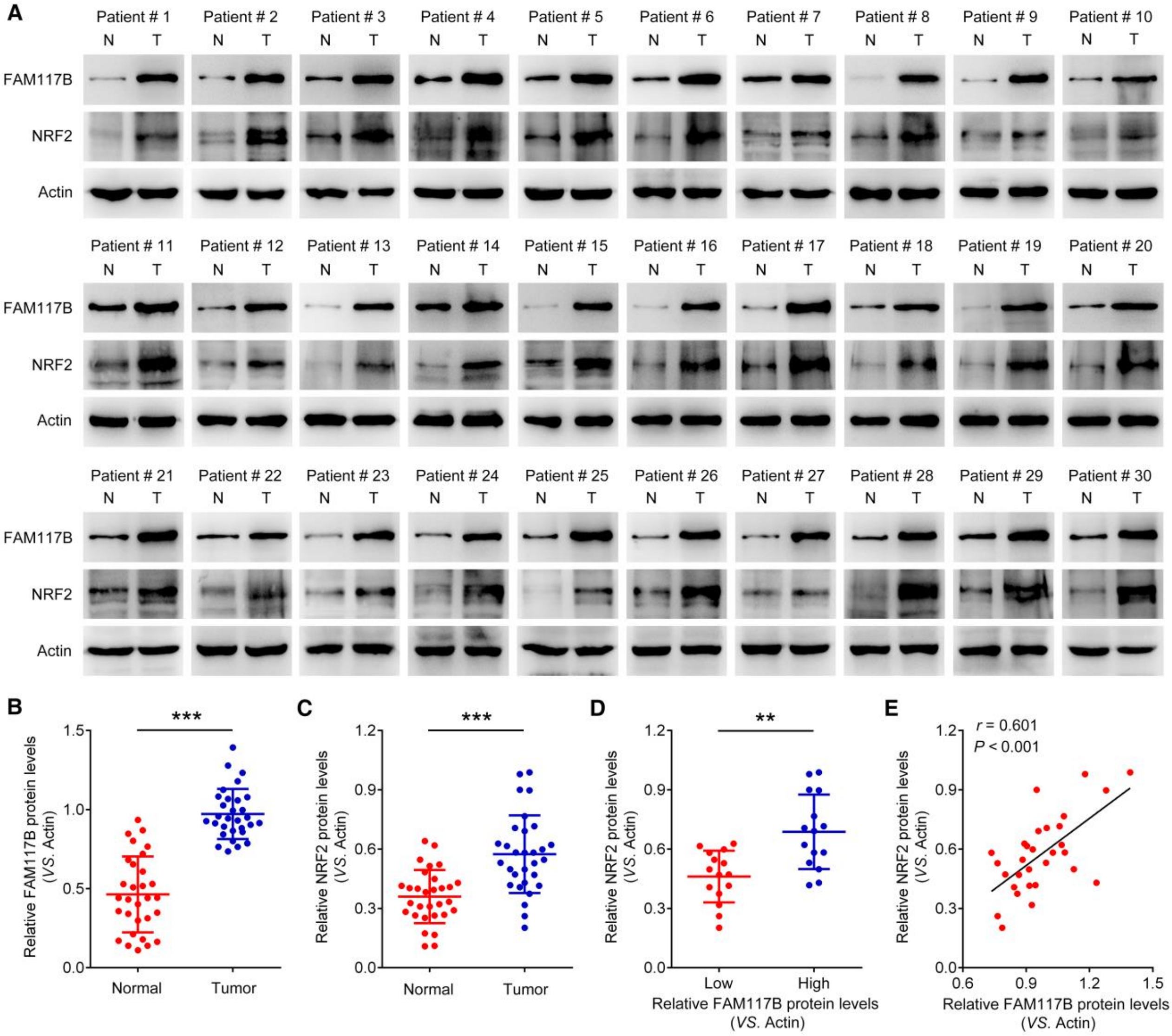

Supplemental Figure 13, related to Figure 9. FAM117B and NRF2 are both overexpressed in gastric cancer tissues and their co-overexpression represents a factor for poor prognosis. (**A-C**) The protein levels of FAM117B and NRF2 in tumor tissues (n = 30) and matched adjacent normal tissues of gastric cancer patients (n = 30). (**D**) The protein levels of NRF2 in tumor tissues with low (n = 15) or high (n = 15) FAM117B protein level. (**E**) Spearman correlation analysis of FAM117B and NRF2 protein levels in tumor tissues (n = 30). Data are shown as mean ± SD. $^{**}P < 0.01$ and $^{***}P < 0.001$ mean significant difference. The statistical significance was calculated using 2-tailed unpaired Student's t test or 2-tailed Spearman test.

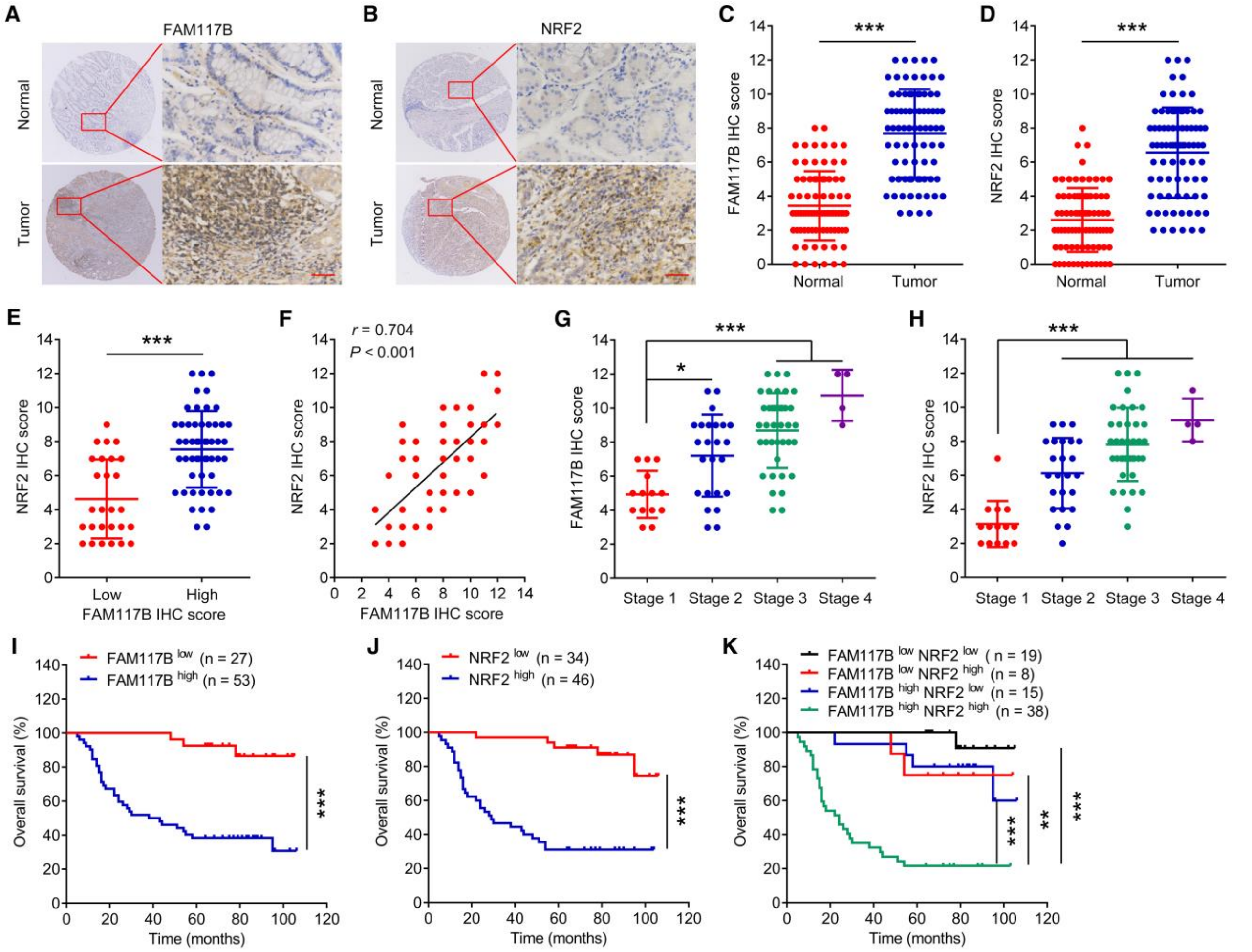

Supplemental Figure 14, related to Figure 9. FAM117B and NRF2 are both overexpressed in gastric cancer tissues and their co-overexpression represents a factor for poor prognosis. (**A-D**) The FAM117B and NRF2 IHC scores of tumor tissues (n = 80) and adjacent normal tissues (n = 80) in AFB cohort. Scale bar = 50 μm. (**E**) The IHC scores of NRF2 in tumor tissues with low (n = 27) or high (n = 53) FAM117B IHC score. (**F**) Spearman correlation analysis of FAM117B and NRF2 IHC scores in tumor tissues (n = 80). (**G** and **H**) The IHC scores of FAM117B and NRF2 in tumor tissues with clinical stage 1 (n = 14), stage 2 (n = 24), stage 3 (n = 38), and stage 4 (n = 4). (**I-K**) Kaplan-Meier survival analysis. Data are shown as mean ± SD. *P < 0.05, **P < 0.01, and ***P < 0.001 mean significant difference. The statistical significance was calculated using 2-tailed unpaired Student's t test (2 groups), one-way ANOVA, followed by Tukey's test (more than 2 groups), 2-tailed Spearman test, or log-rank test.

Supplemental Table 1. IC_{50} values of 5-FU and oxaliplatin in gastric cancer cells transfected with shControl, shFAM117B #1, shFAM117B #2, vector, and FAM117B. Data are shown as mean ± SD, n = 3 independent experiments.

Group	5-FU (IC_{50}, μM)			Oxaliplatin (IC_{50}, μM)		
	HGC-27	AGS	SNU-668	HGC-27	AGS	SNU-668
shControl	64.85 ± 4.7	66.41 ± 4.61	55.89 ± 4.79	7.72 ± 0.44	7.44 ± 0.49	7.36 ± 0.56
shFAM117B #1	21.24 ± 2.02	24.88 ± 2.83	19.91 ± 3.36	2.56 ± 0.2	3.46 ± 0.3	2.57 ± 0.25
shFAM117B #2	20.14 ± 2.75	22.45 ± 2.92	20.15 ± 2.38	2.45 ± 0.16	3.25 ± 0.39	2.38 ± 0.28
Vector	65.35 ± 5.06	63.09 ± 4.7	53.12 ± 5.86	7.55 ± 0.6	7.2 ± 0.65	7.62 ± 0.54
FAM117B	151.56 ± 10.72	127.12 ± 9.22	113.76 ± 8.07	17.29 ± 1.92	15.91 ± 1.69	13.43 ± 2.17

Supplemental Table 2. IC_{50} values of 5-FU and oxaliplatin in gastric cancer cells transfected with shControl, shFAM117B #2, and shFAM117B #2 + FAM117B. Data are shown as mean ± SD, n = 3 independent experiments.

Group	5-FU (IC_{50}, μM)		Oxaliplatin (IC_{50}, μM)	
	HGC-27	AGS	HGC-27	AGS
shControl	62.47 ± 4.85	65.37 ± 3.97	7.67 ± 0.38	7.48 ± 0.52
shFAM117B #2	20.88 ± 1.74	22.72 ± 2.38	2.49 ± 0.15	3.25 ± 0.28
shFAM117B #2 + FAM117B	45.78 ± 3.65	52.59 ± 4.79	6.73 ± 0.47	6.61 ± 0.58

Supplemental Table 3. IC_{50} values of 5-FU and oxaliplatin in gastric cancer cells transfected with vector, HA-FAM117B-WT, HA-FAM117B-M1, HA-FAM117B-M2, HA-FAM117B-M3, HA-FAM117B-M4, and HA-FAM117B-M5. Data are shown as mean ± SD, n = 3 independent experiments.

Group	5-FU (IC_{50}, μM)		Oxaliplatin (IC_{50}, μM)	
	HGC-27	AGS	HGC-27	AGS
Vector	63.92 ± 3.95	59.94 ± 7.1	7.29 ± 0.43	7.17 ± 0.43
HA-FAM117B-WT	148.19 ± 9.27	126.74 ± 10.77	15.5 ± 1.01	14.71 ± 1.28
HA-FAM117B-M1	65.68 ± 5.46	58.04 ± 8.07	7.38 ± 0.57	7.22 ± 0.37
HA-FAM117B-M2	62.74 ± 4.55	61.68 ± 4.96	7.23 ± 0.53	7.31 ± 0.36
HA-FAM117B-M3	61.94 ± 5.63	58.35 ± 6.51	7.15 ± 0.38	7.43 ± 0.56
HA-FAM117B-M4	64.17 ± 4.66	60.92 ± 4.52	7.34 ± 0.5	7.27 ± 0.39
HA-FAM117B-M5	62.33 ± 5.24	59.93 ± 6.06	7.18 ± 0.32	7.09 ± 0.59

Supplemental Table 4. IC_{50} values of 5-FU and oxaliplatin in gastric cancer cells transfected with shControl + Vector, shControl + FAM117B, shNRF2 + Vector, and shNRF2 + FAM117B. Data are shown as mean ± SD, n = 3 independent experiments.

Group	5-FU (IC_{50}, μM)			Oxaliplatin (IC_{50}, μM)		
	**HGC-27**	**AGS**	**SNU-668**	**HGC-27**	**AGS**	**SNU-668**
shControl + Vector	63.28 ± 4.85	61.89 ± 5.37	54.3 ± 4.2	7.37 ± 0.51	7.29 ± 0.57	7.59 ± 0.45
shControl + FAM117B	147.93 ± 7.58	130.13 ± 9.58	119.16 ± 9.05	16.02 ± 1.5	14.06 ± 2.15	14.38 ± 1.26
shNRF2 + Vector	20.68 ± 1.12	19.14 ± 1.88	18.08 ± 2.26	2.2 ± 0.24	2.61 ± 0.15	2.26 ± 0.19
shNRF2 + FAM117B	21.05 ± 1.97	20.69 ± 1.06	19.57 ± 1.98	2.29 ± 0.27	2.72 ± 0.21	2.33 ± 0.25

Supplemental Table 5. Cox regression analysis of risk factors for cancer-related death in two cohorts.

Variables	SOB cohort				AFB cohort			
	Univariate analysis		Multivariate analysis		Univariate analysis		Multivariate analysis	
	HR (95% CI)	*P*	HR (95% CI)	*P*	HR (95% CI)	*P*	HR (95% CI)	*P*
Sex								
Male	1				1			
Female	0.87 (0.45, 1.68)	0.676			1.07 (0.53, 2.18)	0.842		
Age								
< 60 years	1				1			
≥ 60 years	0.84 (0.45, 1.58)	0.59			1.08 (0.56, 2.09)	0.811		
Tumor size								
< 6 cm	1				1		1	
≥ 6 cm	1.46 (0.62, 3.44)	0.392			3.64 (1.88, 7.04)	< 0.001	1.43 (0.68, 3)	0.35
Clinical stage								
1-2	1		1		1		1	
3-4	3.03 (1.46, 6.31)	0.003	1.27 (0.33, 4.86)	0.729	5.23 (2.37, 11.56)	< 0.001	4.9 (0.77, 31.03)	0.092
T status								
T1-T2	1				1			
T3-T4	3.07 (0.95, 9.91)	0.061			1.91 (0.87, 4.18)	0.106		
N status								
N0-N1	1		1		1		1	
N2-N3	3.07 (1.55, 6.08)	0.001	1.66 (0.47, 5.82)	0.431	4.42 (2.12, 9.21)	< 0.001	0.4 (0.08, 2.15)	0.288
M status								
M0	1		1		1		1	
M1	6.89 (2, 23.72)	0.002	3.78 (1.08, 13.17)	0.037	4.46 (1.67, 11.9)	0.003	1.45 (0.53, 4)	0.473
Protein IHC score								
$FAM117B^{low}NRF2^{low}$	1		1		1		1	
$FAM117B^{low}NRF2^{high}$	2.74 (0.17, 43.85)	0.476	2.6 (0.16, 42.08)	0.501	4.86 (0.44, 53.66)	0.197	2.43 (0.19, 31.49)	0.496
$FAM117B^{high}NRF2^{low}$	3.82 (0.35, 42.21)	0.274	2.78 (0.25, 31.35)	0.409	5.06 (0.57, 45.35)	0.147	3.83 (0.41, 35.49)	0.238
$FAM117B^{high}NRF2^{high}$	16.8 (2.31, 122.42)	0.005	11.6 (1.55, 87.02)	0.017	33.77 (4.57, 249.84)	0.001	18.9 (2.37, 150.52)	0.006

Supplemental Table 6. The sequences of primers used in this study.

Gene	Forward (5′-3′)	Reverse (5′-3′)
NRF2	CAGCTTTTGGCGCAGACATT	GACTGGGCTCTCGATGTGAC
HO-1	CTTTCAGAAGGGCCAGGTGA	GTAGACAGGGGCGAAGACTG
NQO1	GGTTTGGAGTCCCTGCCATT	TTGCAGAGAGTACATGGAGCC
PRDX1	CCCACGGAGATCATTGTT	CGAGATGCCTTCATCAGCCT
PRDX2	GAAGCTGTCGGACTACAAAGG	TCGGTGGGGCACACAAAAG
PRDX3	GCCGTTGTCAATGGAGAGTT	TCCACTGAGACTGCGACAAC
PRDX4	AGAGGAGTGCCACTTCTACG	GGAAATCTTCGCTTTGCTTAGGT
SRXN1	CAGGGAGGTGACTACTTCTACTC	CAGGTACACCCTTAGGTCTGA
SOD2	TGGGGTTGGCTTGGTTTCAA	GGAATAAGGCCTGTTGTTCCTTG
CAT	CGGAGATTCAACACTGCCAATG	TTCTTGACCGCTTTCTTCTGGA
GCLC	GGACAAGAATACACCATCTCCA	ATACTGCAGGCTTGGAATGTC
GCLM	GGGAACCTGCTGAACTGG	CTGGGTTGATTTGGGAACTC
GSR	AGGAGCTGGAGAACGCTGGC	CAATGGCCCAGAGCAGGCA
GSTA2	GGCTGCAGCTGGAGTAGAGT	AAGGCAGGGAAGTAGCGATT
GSTA4	GGCAGCAAGGCCCAAGCTCCACT	GGCCTAAAGATGTTGTAGACGG
GSTM2	ACA ACCTGTGCGGGGAATC	AGCTTCAGCATTTCAGGGAGTG
GSTM3	GACTTTCCTAATCTGCCCTACCTC	TTCTTCTTCAGTCTCACCACACAT
AKR1B1	TATTCACTGGCCGACTGGCTTTA	GAACCACATTGCCCGACTCA
AKR1B10	GCAGGACGTGAGACTTCTACC	ATCCTGCATCAATGGCCACC
AKR1C1	TTCATGCCTGTCCTGGGATTT	CTGGCTTTACAGACACTGGAAAA
AKR1C3	GGATTTGGCACCTATGCACCTC	CTATATGGCGGAACCCAGCTTCTA
ALDH1A1	GCACGCCAGACTTACCTGTC	CCTCCTCAGTTGCAGGATTAAAG
ALDH3A1	ACTGGGCGTGGTCCTCGTCATTGG	GTGAGGATGGTGGGGGCTATGTAG
ABCC1	CTCTATCTCTCCCGACATGACC	AGCAGACGATCCACAGCAAAA
ABCC2	CTTGGGCTTCCTATGGCTCC	ATCGAACAGCAGGGACTGTG
ABCC3	CAGAGAAGGTGCAGGTGACA	CTAAAGCAGCATAGACGCCC
ABCC5	GTTCAGGAGAACTCGACCGTTGG	TTTGGAAGTAGTCCGGATGGGCTT
IDH1	TGCAAAAATATCCCCCGGCT	TACATCCCCATGGCAACACC
ME1	CTGCCTGTCATTCTGGATGT	ACCTCTTACTCTTCTCTGCC
PGD	ATTCTCAAGTTCCAAGACACCG	GTGGTAAAACAGGGCATGGGA
GAPDH	CTGACTTCAACAGCGACACC	TGCTGTAGCCAAATTCGTTGT
